# Single-cell RNA sequencing reveals functional heterogeneity of glioma-associated brain macrophages

**DOI:** 10.1038/s41467-021-21407-w

**Published:** 2021-02-19

**Authors:** Natalia Ochocka, Pawel Segit, Kacper Adam Walentynowicz, Kamil Wojnicki, Salwador Cyranowski, Julian Swatler, Jakub Mieczkowski, Bozena Kaminska

**Affiliations:** 1 Laboratory of Molecular Neurobiology, Nencki Institute of Experimental Biology of the Polish Academy of Sciences, Warsaw, Poland; 2 Postgraduate School of Molecular Medicine, Medical University of Warsaw, Warsaw, Poland; 3 Laboratory of Cytometry, Nencki Institute of Experimental Biology of the Polish Academy of Sciences, Warsaw, Poland

**Keywords:** Cancer, Computational biology and bioinformatics, Immunology, Molecular biology

## Abstract

Microglia are resident myeloid cells in the central nervous system (CNS) that control homeostasis and protect CNS from damage and infections. Microglia and peripheral myeloid cells accumulate and adapt tumor supporting roles in human glioblastomas that show prevalence in men. Cell heterogeneity and functional phenotypes of myeloid subpopulations in gliomas remain elusive. Here we show single-cell RNA sequencing (scRNA-seq) of CD11b^+^ myeloid cells in naïve and GL261 glioma-bearing mice that reveal distinct profiles of microglia, infiltrating monocytes/macrophages and CNS border-associated macrophages. We demonstrate an unforeseen molecular heterogeneity among myeloid cells in naïve and glioma-bearing brains, validate selected marker proteins and show distinct spatial distribution of identified subsets in experimental gliomas. We find higher expression of MHCII encoding genes in glioma-activated male microglia, which was corroborated in bulk and scRNA-seq data from human diffuse gliomas. Our data suggest that sex-specific gene expression in glioma-activated microglia may be relevant to the incidence and outcomes of glioma patients.

## Abstract (web summary)

Microglia and peripheral myeloid cells are critically involved in the immunopathology of glioblastoma. Here the authors present single-cell sequencing data that assesses the phenotypic composition of CD11b + myeloid cells from a murine model of glioblastoma and suggest enhanced MHCII transcription which they additionally report from human cases of glioblastoma.

## Introduction

Innate immune cells are abundant in the tumor microenvironment (TME) and play pivotal role in tumor progression and modulation of responses to therapy^1^. High numbers of macrophages within the TME have been associated with poor prognosis in many cancers, because those tumor-educated cells suppress antitumor immunity, stimulate angiogenesis, and promote tumor invasion^2^. The central nervous system (CNS) is equipped with resident innate immune cells: microglia, and CNS border-associated macrophages (BAMs) that migrate to the CNS during the prenatal life and maintain a long-lasting population. In malignant gliomas, both local microglia and circulating monocytes (Mo) migrate to the TME and differentiate into tumor supporting cells, commonly referred to as glioma-associated microglia and macrophages (GAMs). Reliable identification of specific subpopulations is hampered by a shortage of specific markers^3^. Transcriptome profiling of bulk CD11b^+^ cells isolated from human glioblastomas (GBMs) and rodent gliomas showed a mixture of protumorigenic and antitumorigenic phenotypes, and did not reveal consistent markers and pathways^4–6^. Recent reports showed that GAMs consist of diverse cell populations with likely distinct roles in tumor progression^7–10^. Dissecting the TME composition and functional heterogeneity of tumor-infiltrating immune cells would extend the understanding of glioma immune microenvironment, and allow to modulate functions of distinct subpopulations for therapeutic benefits.

Sex differences in incidence (male-to-female ratio of 1.6:1), transcriptomes, and patient outcomes in adult GBM patients have been previously reported^11^. Sex-specific disease outcomes can be related to immune functions, because the efficacy of cancer immunotherapy in humans was shown to be largely depending on sex, with better outcomes in males^12^. In naïve mice, male microglia show enrichment of inflammation and antigen presentation-related genes, whereas female microglia have higher neuroprotective capacity^13,14^. Until now, sex differences have been largely unexplored in animal studies on glioma immunobiology.

Here, we used single-cell RNA sequencing (scRNA-seq) to decipher the composition and functions of GAMs in murine experimental GL261 gliomas grown in male and female mice. We demonstrate distinct transcriptional programs of microglia (MG), monocytes/macrophages (Mo/MΦ), and CNS BAMs. The identified MG and Mo/MΦ signature markers allow for a separation of these cells within glioma TME. Intracranial gliomas activate similar transcriptional networks in MG and Mo/MΦ present in TME. However, transcriptional responses of Mo/MΦ are more pronounced and associated with activation of immunosuppressive genes. In males, MG and a fraction of Mo/MΦ infiltrating gliomas show higher expression of the major histocompatibility complex II (MHCII) genes, suggesting stronger activation of male MG. Altogether, this study demonstrates considerable cellular and functional heterogeneity of myeloid cells in TME and is suggestive of sex-specific differences in responses of myeloid cells to gliomas.

## Results

### Single-cell RNA-seq identifies myeloid cells with distinct expression profiles among CD11b^+^ cells from naive and glioma-bearing brains

**Fig. 1:**
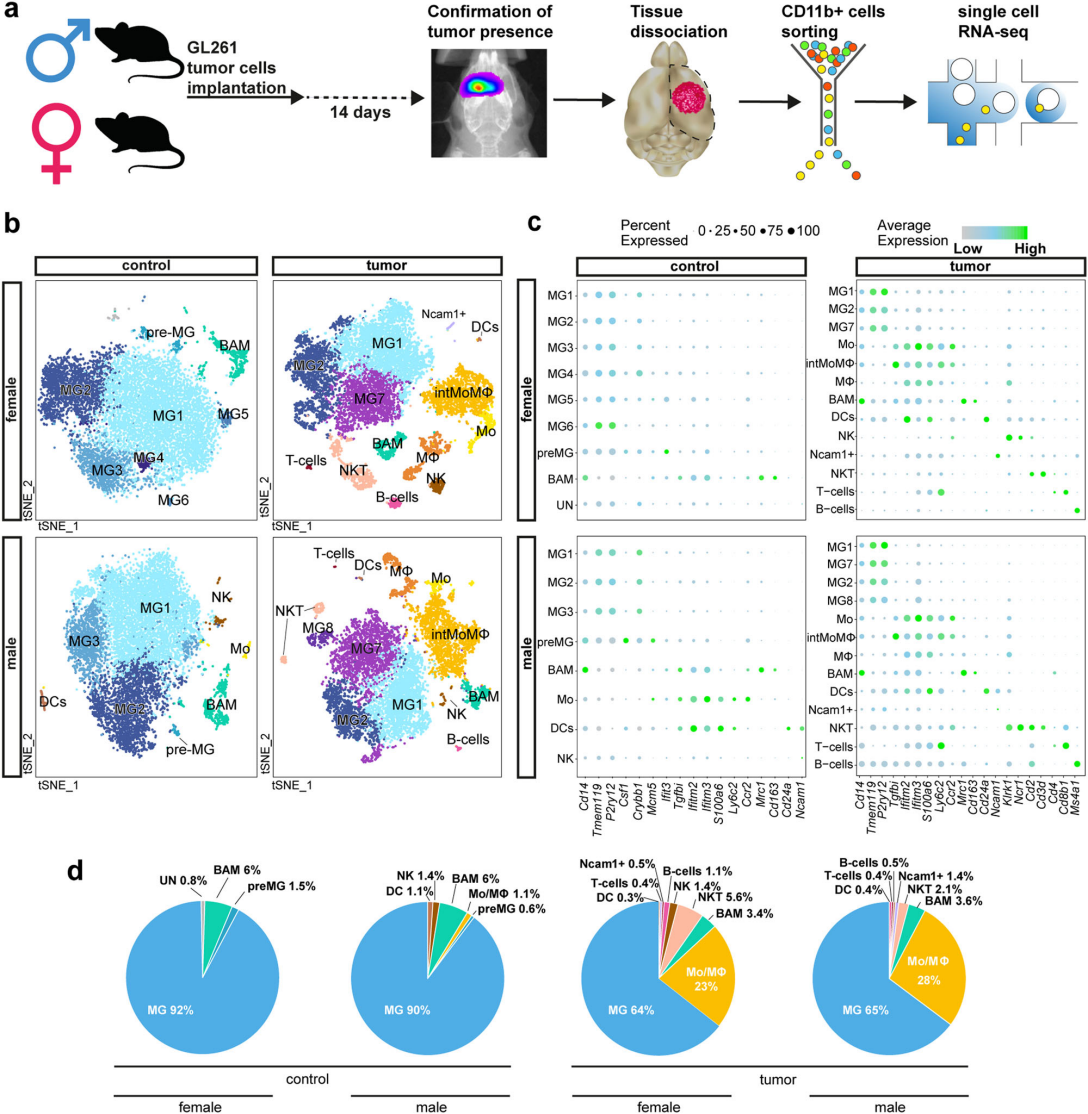
Identification of immune cell populations in control and tumor-bearing brain hemispheres. **a** Scheme of the experimental workflow. The used brain image was modified from Database Center for Life Science. **b** *t*-SNE plot demonstrating clustering obtained for each group (female control, female tumor, male control, and male tumor), two biological replicates were combined. Clusters annotations: MG microglia, preMG premature microglia, Mo monocytes, intMoMΦ intermediate monocyte–macrophage, MΦ macrophages, BAM CNS border-associated macrophages, DCs dendritic cells, Ncam1+ Ncam1-positive cells, NK natural killer cells, NKT natural killer T cells, B cells B lymphocytes, T cells T lymphocytes. **c** Expression of “signature” genes selected from the immune marker panel for identification of a cluster cell type (Supplementary Table 1). **d** Pie charts demonstrating distribution of the identified cell types across samples.

We employed a murine orthotopic GL261 glioma model, because tumors established from GL261 cells recapitulate many characteristics of human GBMs and are frequently used in studies of glioma immunology, immunotherapy, and in preclinical studies^15^. To assess the heterogeneity of GAMs in GL261 gliomas, we performed scRNA-seq on CD11b^+^ cells sorted from naïve and tumor-bearing brains of male and female mice (two replicates per group, two pooled mice per replicate; Fig. 1a). We used nave brains as controls, because scRNA-seq data for CD11b^+^ cells sorted from brains of naïve and sham-implanted animals did not indicate that the surgical procedure affects identified cell populations, their proportions, and gene expression (Supplementary Fig. 1e–h). The tumor-bearing animals were sacrificed 14 days post implantation. This time point corresponds to a presymptomatic stage of tumorigenesis, when GL261 tumors are restricted to a single hemisphere, show a substantial infiltration of peripheral Mo/MΦ^16^, and no signs of necrosis that could affect perfusion (Supplementary Fig. 1a–d). Using fluorescence-activated cell sorting, we sorted CD11b^+^ cells with a particularly high purity (>96%) and viability (~95%; Supplementary Fig. 1d). We did not detect differences in the tumor size between sexes at this stage of the tumor growth (Supplementary Fig. 1e).

To resolve the molecular profiles of CD11b^+^ cells, we performed scRNA-seq. After quality control and adjusting for technical noise, single-cell transcriptomic profiles for 40,401 cells and 14,618 genes were selected for the analysis (see “Methods” section). We visually inspected the transcriptomic diversity of computed clusters, projecting the data onto two dimensions by *t*-distributed stochastic neighbor embedding (*t*-SNE; Fig. 1b). To characterize the cell identity of the obtained clusters, we applied the immune cell marker panel (Fig. 1c) created with the literature-based markers (Supplementary Table 1)^3,7,8,10,17–30^. The cell identities were inferred by identifying significantly overexpressed genes in each cluster.

Unsupervised clustering of each group demonstrated a similar number of clusters between sexes (Fig. 1b). To avoid over-fitting, we used the same parameters for all cluster analyses (see “Methods” section) and this might have resulted in different stratification of the analyzed conditions. However, this analysis was done primarily to select cells for further processing.

For naïve female and male CD11b^+^ cells, nine and eight clusters were obtained, respectively. Gene expression profiles underlying a specific cluster could reflect different functions of contained cells or their different origin from various brain structures. To dissect functional meaning of gene expression underlying microglial clusters (MG), we explored the available information on microglial phenotypes^20,21,31,32^. The predominant cluster MG1 characterized by relatively high expression of microglia-enriched genes (*Crybb1*, *Cst3*, *P2ry12*, and *Pros1*)^31^ may reflect a subpopulation of homeostatic microglia (Hom-MG). Cluster MG2 is characterized by a high expression of immediate early genes (*Jun*, *Junb*, *Jund*, *Fos*, *Egr1*, *Klf6*, and *Aft3*) encoding transcription factors, and may encompass a subpopulation of transcriptionally active cells (Supplementary Data 1). MG2 shows also an increased expression of *Nfkbia* encoding an NFκB inhibitor alpha. Cluster MG3 is marked by high expression of genes coding for signaling inhibitors: *Bmp2k* and transcriptional repressors: *Bhlhe41*, *Ncoa3*, and *Notch2* (Supplementary Data 1). The transcription factors Bhlhe40 and Bhlhe41 directly repress the expression of lineage‐inappropriate genes in alveolar macrophages^33^. *Ncoa3* in association with nuclear receptors represses expression of inflammation mediators and activates genes encoding anti-inflammatory mediators^34^. Three microglial clusters represented by a smaller cell number: MG4, MG5, and MG6 were identified in female naïve CD11b^+^ cells. MG4 did not show cluster-specific genes. MG5 showed increased expression of myelin specific genes: *Plp1*, *Pltp*, and *Mbp*, which are found in microglia with increased myelin uptake^32^. Among top highly expressed genes in MG6, we found *Cd63* and *Cd9* encoding proteins that are enriched in extracellular vesicles^35^.

In both male and female CD11b^+^ cells from naïve brains, we identified preMG cluster characterized by an increased expression of microglial genes (*Tmem119*, *P2ry12*, and *Crybb1*) and genes characteristic for their premature state (*Csf1*, *Mcm5*, and *Ifit3*)^21^ (Fig. 1c). PreMG upregulated genes encoding a cysteine protease inhibitor (*Cst7*), cytokines (*Mif* and *Csf1*), chemokines (*Ccl12*, *Ccl3*, and *Ccl4*), genes involved in a response to interferon (*Ifit1*, *Ifit3*, *Ifit3b*, *Ifitm3*, and *Irf7*), and genes implicated in a ubiquitin-like process of ISG-ylation (*Isg15* and *Usp18*) that are activated during inflammation (Supplementary Data 1). These microglia could represent surveying cells fitted to rapidly respond to homeostasis dysfunction.

Among CD11b^+^ cells from tumor-bearing hemispheres, we identified 13 clusters for both sexes. In TME-infiltrating MG, besides the presence of previously described clusters MG1-2, we found a MG7 cluster characterized by increased expression of genes encoding the components of MHC class I (*B2m*, *H2-D1*, and *H2-K1*) and MHC class II (*H2-Oa* and *H2-DMa)*, *Bst2 and Lgals3bp*, upregulation of which has been reported in disease-associated microglia^36,37^, and *Ccl12* encoding a cytokine critical for CCR2^+^ Mo recruitment^38^. Among TME-infiltrating male MG, we found cluster MG8 characterized by a high expression of genes encoding proliferation-related proteins (*Stmn1*, *Tubb5*, *Tuba1b*, *Cdk1*, and *Top2a*), which is consistent with the observed proliferation of MG within glioma TME, as previously reported^6,39^. MG from tumor bearing animals show also upregulation of *Timp2*, *Serpine2*, *Cst7*, and *Ctsd*, genes encoding proteases or their modulators participating in reorganization of extracellular matrix, which may reflect invasion supporting properties. The identified MG clusters may represent a transient, intermediate activation states of microglia.

In naïve brains, MG comprised the vast majority of all sorted cells (91% in females, 90% in males), whereas BAMs constituted 6% of cells in both sexes (Fig. 1d). Amongst CD11b^+^ cells from male controls, we found a small subset of Mo (*Ly6c2*^+^, and *Ccr2*^+^), natural killer (NK, *Ncam1*^+^), and dendritic cells (DC, *Cd24a*^+^).

In tumor-bearing brains, MG were still the most abundant cell population (64% in females, 65% in males), although their proportion decreased due to infiltration of Mo/MΦ, forming the second main myeloid cell population of the TME (23% in females, 28% in males; Fig. 1d). For both sexes, we identified three clusters of infiltrating Mo/MΦ that could be further characterized by an inflammatory monocyte signature—Mo (*Ly6c2*^high^, *Ccr2*^high^, and *Tgfbi*^low^), an intermediate state of monocyte and macrophage signature—intMoMΦ (*Ly6c2*^high^ and *Tgfbi*^high^), and a differentiated macrophage signature—MΦ (*Ly6c2*^low^, *Ifitm2*^high^, *Ifitm3*^high^, and *S100a6*^high^; Fig. 1c). These results demonstrate dynamic changes in Mo/MΦ-infiltrating gliomas. We found minor populations of NK cells, DCs, NKT cells, and a marginal fraction of B and T cells. CD11b^+^ is not expressed on lymphocytes, but rare CD11b^+^ lymphocytes (<1%) may appear after activation of the immune response^40,41^. Nevertheless, a vast majority of cells were MG, Mo/MΦ, and BAM.

### Assessment of new and known cell type-specific markers

**Fig. 2:**
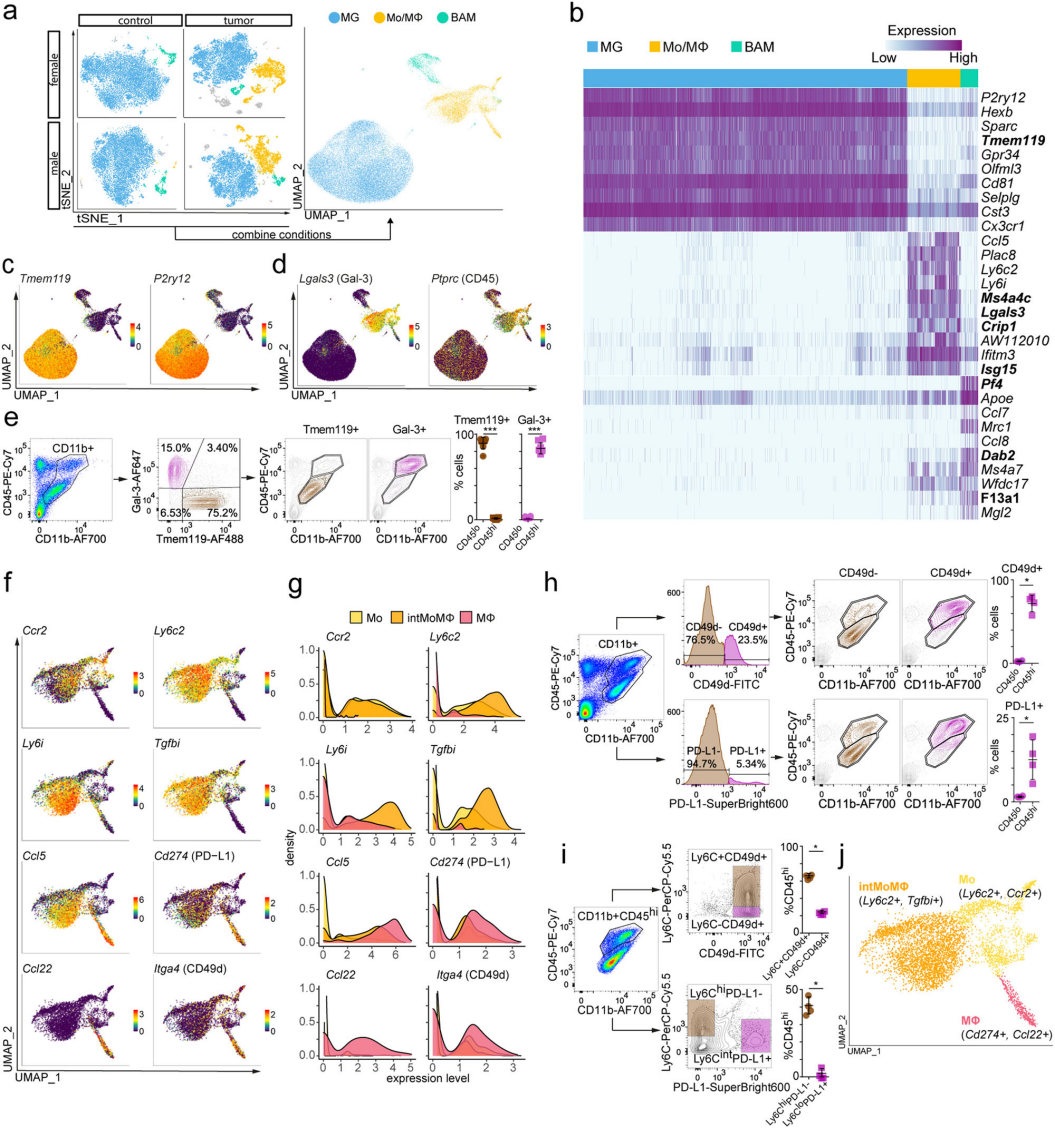
Transcriptomic characterization of main myeloid subpopulations. **a** Projection of cells combined from clusters identified as microglia (MG), monocytes/macrophages (Mo/MΦ), and BAMs from all groups. **b** Top ten differentially expressed genes for the three main identified cell populations, new marker candidates are in bold. **c**, **d** Feature plots depicting genes highly expressed in MG (**c**) and MoMΦ (**d**). **e** Flow cytometric analysis of the distribution of Tmem119 and Gal-3 protein markers within CD11b^+^ cells and projection of Tmem119^+^ and Gal-3^+^ cells onto CD45/CD11b graphs, dot plots demonstrate percentages of Tmem119^+^ and Gal-3^+^ cells within CD45^hi^ and CD45^lo^ groups (*n* = 8, 4 males, and 4 females, two-sided Mann–Whitney *U* test, mean ± SD, *** < 0.001, Tmem119 Pv = 0.0002, Gal-3 Pv = 0.0002). **f** Feature plots depicting distribution of the expression of genes discriminating monocytes (Mo), monocyte–macrophage intermediate (intMoMΦ), and macrophage (MΦ) subpopulations. **g** Density plots demonstrating the expression level of markers discriminating the Mo/MΦ subpopulations. **h** Flow cytometry analysis of CD49d and PD-L1 proteins within CD11b^+^ cells and their projection onto CD11b/CD45 graphs, dot plots demonstrate percentages of CD49d^+^ and PD-L1^+^ cells within CD45^hi^ and CD45^lo^ groups (*n* = 4, 2 males, and 2 females; two-sided Mann–Whitney *U* test, mean ± SD, * < 0.05, CD49d Pv = 0.0286, PD-L1 Pv = 0.0286). **i** Flow cytometry analysis of the distribution of the markers discriminating Mo/MΦ subpopulations within CD11b^+^CD45^hi^ cells, dot plots demonstrate percentage of CD11b^+^CD45^hi^ cells that belong to the defined populations (*n* = 4, 2 males, and 2 females; two-sided Mann–Whitney *U* test, mean ± SD, * < 0.05, Ly6C CD49d Pv = 0.0286, Ly6C PD-L1 Pv = 0.0286). **j** UMAP plot showing clusters of Mo/MΦ subpopulations.

To identify the molecular features that distinguish naive and tumor-associated myeloid cells, we performed further analyses on major cell subpopulations. From all the conditions and replicates, we extracted only the cells identified as MG, Mo/MΦ, and BAMs. In all conditions, both scRNA-seq replicates similarly contributed to these results (Supplementary Fig. 2), demonstrating good reproducibility. The combined three cell subpopulations, projected on the two-dimensional space using a Uniform Manifold Approximation and Projection (UMAP) algorithm, formed three separate groups (Fig. 2a and Supplementary Fig. 3). This observation demonstrates a predominance of a biological signal over technical artifacts or batch effects. To confirm cell identities, we performed differential expression analyses between three subpopulations of CD11b^+^ cells. Among the most highly upregulated genes in each group (see “Methods” section for details of differential gene expression analysis), we found the well-known microglial genes—*P2ry12*, *Sparc*, *Tmem119*, *Gpr34*, *Selplg*, and *Cx3cr1* (refs. ^19,42^) in MG, Mo—*Ly6i* and *Ly6c2*, and MΦ genes—*Ifitm3* (ref. ^10^) in Mo/MΦ, and BAM genes—*Apoe*, *Ms4a7*, and *Mrc1* (ref. ^43^) in BAMs (Fig. 2b). The expression of *Tmem119*, *Cx3cr1*, *P2ry12*, *Gpr34*, *Olfml3*, and *Sparc* was enriched only in MG (Fig. 2b and Supplementary Fig. 5a). Other genes expressed at a high level in MG were also highly expressed in BAMs (*Cd81*), BAMs and Mo/MΦ (*Hexb* and *Cst3*) or were found only in a fraction of cells (*P2ry13* gene was expressed by <75% of MG cells; Supplementary Fig. 5a). For Mo/MΦ, we found enriched expression of previously reported genes, such as *Ifitm2*, *S100a6*, and *S100a11* (ref. ^10^), as well as novel genes, namely *Ms4a4c*, *Lgals3*, *Crip1*, and *Isg15* (Fig. 2b and Supplementary Fig. 5b). *Ifitm3* was highly expressed by the Mo/MΦ population, but appeared in a substantial fraction of MG, showing its low specificity in Mo/MΦ within glioma TME. Highly expressed genes in BAMs were *Apoe* and *Ms4a7*, recently proposed as markers of CNS border macrophages^43^. However, we found these genes also highly expressed by Mo/MΦ, suggesting that *Apoe* and *Ms4a7* are not exclusive for BAMs in TME. *Mrc1* showed high expression restricted to BAMs. In addition, we found *Pf4*, *Dab2*, *and F13a1* highly and specifically expressed by BAMs (Fig. 2b and Supplementary Fig. 5c).

We aimed to identify markers for separation of MG and Mo/MΦ in TME. From the top differentially expressed genes (ranked by the average log fold-change value, all with adjusted (Bonferroni correction) *p* value < 10^−100^) in the MG and Mo/MΦ groups (Fig. 2b), we selected candidate genes with enriched expression in a majority of cells in the group of interest—*Tmem119* (MG) and *Lgals3* (Mo/MΦ; Fig. 2c, d). *Tmem119* was proposed as a MG marker by Bennet et al.^19^, who confirmed its utility in CNS inflammation and nerve injury. *Lgals3* encodes galectin-3 (Gal-3), a lectin involved in tumor immunosuppression^44^. Gal-3 is produced and secreted by MΦ, regulates IL-4 induced alternative macrophage activation^45^ and acts as Mo/MΦ chemoattractant.

We assessed Tmem119 and Gal-3 expression in CD11b^+^ cells from tumor-bearing hemispheres by flow cytometry at day 14 post implantation (Fig. 2e). Brains were mechanically processed and dissociated enzymatically with DNase I to preserve Tmem119 surface expression  (see Supplementary Fig. 6a and “Methods” section). Gal-3 and Tmem119 allowed for the discrimination of two populations: Tmem119^+^Gal-3^−^ (75.2% of cells) and Tmem119^−^Gal-3^+^ (15.0% of cells), whereas Tmem119^+^Gal-3^+^ population was minor (Fig. 2e). These results correspond to scRNA-seq analysis, in which 60% and 21.9% of CD11b^+^ cells expressed only *Tmem119* or *Lgals3*, respectively. We assessed Tmem119 and Gal-3 expression in CD11b^+^CD45^lo^ and CD11b^+^CD45^hi^ cells, in order to compare these marker candidates with the previously used method. Interestingly, the two methods produced similar separation, as 89.5% of CD11b^+^CD45^lo^ cells were Tmem119^+^ and 83.4% of CD11b^+^CD45^hi^ cells were Gal-3^+^ (Fig. 2e).

Among the highly upregulated Mo/MΦ genes, we found candidates enriched in discrete subpopulations of Mo/MΦ. The high *Ly6c2* expression was found in a large cell fraction, which could be further divided into *Ly6c2*^high^*Ccr2*^high^ Mo and *Ly6c2*^high^*Tgfbi*^high^ monocyte/macrophage intermediate cells (intMoMΦ; Fig. 2f,g). The remaining cells resembled differentiated tissue macrophages (MΦ), because they lacked the markers of the cytotoxic Mo (*Ly6c2* and *Ccr2)* and had a strong “macrophage signature” (*Ifitm2*^high^, *S100a6*^high^, and *S100a11*^high^; Supplementary Fig. 5b).

Notably, we found a population of MΦ expressing *Ccl22* and *Ccl5* genes, encoding chemokines important for T-cell recruitment^46,47^ and *Cd274*, a gene encoding an immune checkpoint protein PD-L1 (Fig. 2f, g). Such expression pattern suggests a putative role of these cells in mediating the immunosuppressive response. Flow cytometric analysis confirmed that PD-L1 expression is restricted to the CD11b^+^CD45^high^ population (Fig. 2h). Distribution of Ly6C and PD-L1 among CD45^hi^ population indicates that those proteins denote distinct populations among peripheral myeloid cells infiltrating gliomas: Ly6C^high^PD-L1^−^ intMoMΦ and Ly6C^low^PD-L1^+^ MΦ(Fig. 2i and Supplementary Fig. 6). Thus, we identified genes enriched in the Mo/MΦ subpopulations (Fig. 2j).

We also examined the expression of genes recently proposed as specific markers of Mo/MΦ in gliomas: *Itga4* (ref. ^7^), *Hp*, *Emilin2*, *Sell*, and *Gda*^23^ (Fig. 2f and Supplementary Fig. 5d). The expression of *Itga4* (CD49d) was low and limited mostly to the MΦ subpopulation, resembling differentiated macrophages and expressing a high level of *Cd274* (PD-L1). However, flow cytometric analysis showed that the CD49d protein is expressed by 72.1% of CD11b^+^CD45^hi^ cells (Fig. 2h), out of which 66.2% are Ly6C^hi^ and 24.5% Ly6C^lo^ (Fig. 2i), demonstrating that CD49d protein is expressed in both monocytic and macrophage fraction of bone marrow-derived macrophages (BMDM). CD49d was not found in CD11b^+^CD45^lo^ cells, which corroborated its specificity toward the Mo/MΦ compartment. The expression of *Hp*, *Emilin2*, *Sell*, and *Gda*, the markers suggested in recent meta-analysis of bulk RNA-seq data sets, and validated at RNA and protein levels^23^, was found in the fraction of Mo (Ly6c*2*^hi^ and Ccr2^hi^; Supplementary Fig. 5e). Expression of  *Tgm2* and *Gpnmb*, previously reported as the genes commonly upregulated by GAMs across different glioma animal models and in a bulk RNA-seq of patient-derived samples^3^, was limited to the small fraction of Mo/MΦ (Supplementary Fig. 5e). This observation shows how bulk RNA-seq results may be biased by genes expressed at a high level in a small subset of cells.

Summarizing, we validated the expression of known markers at the single-cell level, and obtained a coherence of selected microglia and BAM markers in our data set with literature data. In contrast, Mo/MΦ in TME showed substantial heterogeneity that is likely related to their differentiation state.

### Distinct gene expression profiles of glioma-associated microglia and monocytes/macrophages

**Fig. 3:**
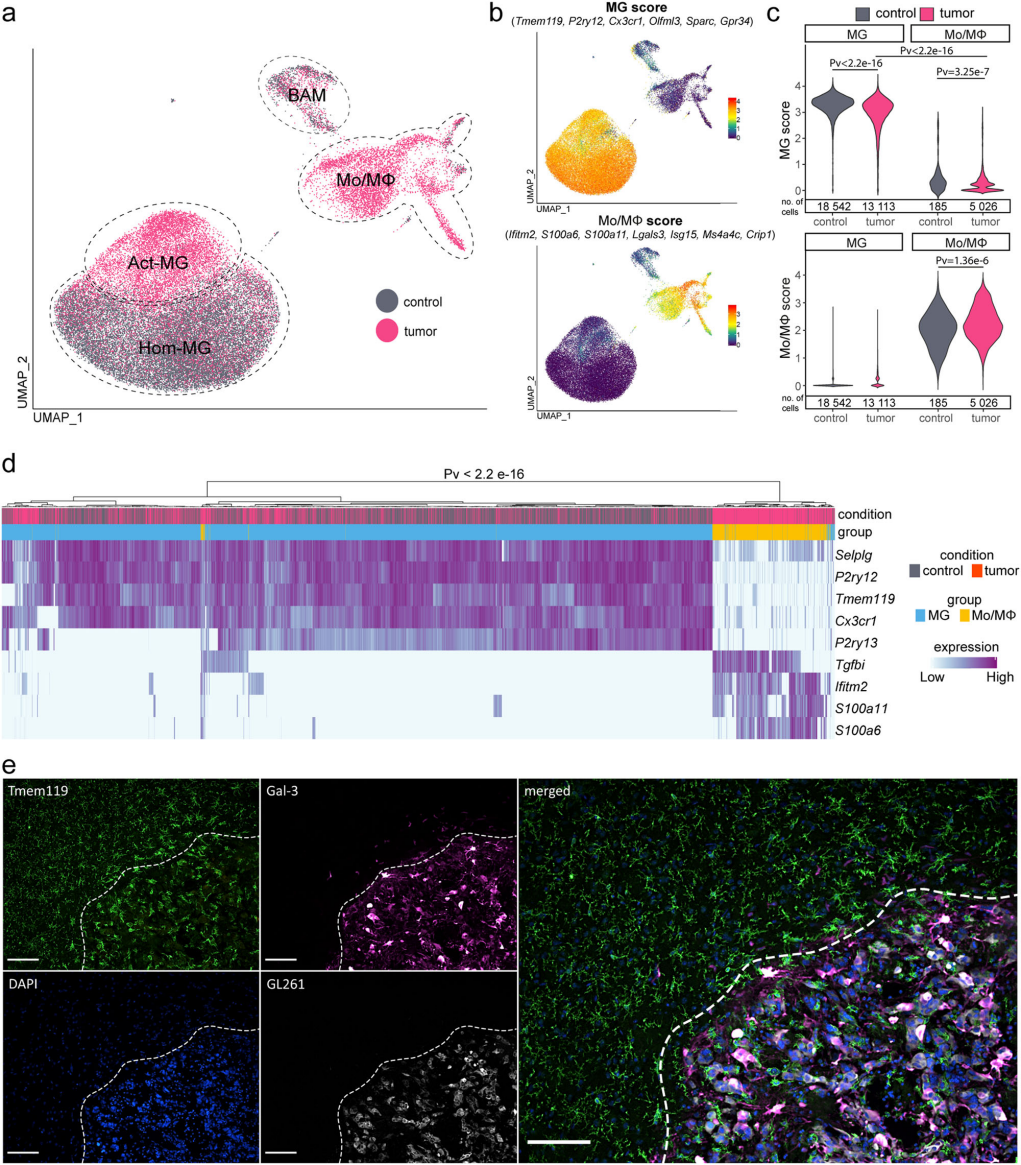
Tumor-derived microglia and macrophages form separate cell populations. **a** UMAP plots demonstrate the distribution of CD11b^+^ cells from naive and tumor-bearing mice. **b** Distribution of MG and Mo/MΦ “signature” gene scores (presented as an average of expression of the selected genes). **c** Density plots of MG and Mo/MΦ scores across MG and Mo/MΦ populations demonstrating no overlap of a specific “signature” between the two cell populations, two-sided Wilcoxon signed-rank test. **d** Cell hierarchical clustering according to the expression of reported macrophage markers demonstrating bimodal cell distribution, two-sided Fisher’s exact test. **e** Immunohistochemical staining for microglia (Tmem119^+^ and Gal-3^−^) and Mo/MΦ (Tmem119^−^ and Gal-3^+^) shows the localization of specific immune cells within the tumor and its surroundings in female animal (for male, see Supplementary Fig. 8); a dashed line marks the tumor edge; scale, 100 μm; the staining was performed for three animals, four sections each, a representative image is shown.

Distribution of cells according to the experimental conditions (naïve vs tumor) revealed separation of functional subgroups of MG. This separation was further supported by the unsupervised clustering that led to clusters either highly enriched in the cells from naïve brains representing Hom-MG or clusters dominated by cells originating from the tumor-bearing hemispheres representing Act-MG (Fig. 3a and Supplementary Fig. 7). This result demonstrates activation of MG within TME. Mo/MΦ cell fraction is composed of the TME-infiltrating Mo/MΦ. BAMs from naive and tumor-bearing brains distributed evenly and did not show any clusters of cells originating predominantly from tumor-bearing hemispheres (Fig. 3a).

Using MG and Mo/MΦ scores (defined as an average of expression levels of genes restricted to and highly expressed in a given population; Fig. 3b), we examined whether microglia and macrophage “signature” gene expression is modified in TME. We found a shift toward the lower “microglia signature” score in MG from the tumor-bearing brains compared to those from the naïve brains (Fig. 3c). Still, the “microglia signature” in MG from the tumor was strong and distinguishable from Mo/MΦ, allowing for clear separation of the two cell populations. Similarly, the “macrophage signature” score was high and distinctive for the Mo/MΦ population. Using selected markers, we performed hierarchical clustering of cells according to the expression of reported microglia and macrophage markers, resulting in clear separation of microglia and Mo/MΦ (Fig. 3d). This observation indicated that the expression of signature genes is retained even under the strong influence of the glioma microenvironment.

We separated distinct CD11b^+^ subpopulations: MG (Tmem119^high^) and Mo/MΦ (Gal-3^high^) by flow cytometry (Fig. 2e). Using immunostaining, we studied spatial localization of those populations in TME. We demonstrate that Tmem119^+^ MG adopt an amoeboid morphology in the tumor proximity and localize abundantly at the tumor edge, whereas Gal-3^+^ Mo/MΦ accumulate mostly within the tumor mass in both female (Fig. 3e) and male animals (Supplementary Fig. 8). This finding confirms previous reports demonstrating that MG occupy the tumor periphery and Mo/MΦ localize mostly within the tumor core^9,48^.

Altogether, we show that MG undergo glioma-induced activation associated with slight reduction of “microglia signature” gene expression, but both MG and Mo/MΦ retain expression of “signature” genes within TME. Staining for Tmem119 and Gal-3 separates MG and Mo/MΦ in murine gliomas.

### Transcriptional networks induced in microglia by glioma are present and more pronounced in infiltrating monocytes/macrophages

**Fig. 4:**
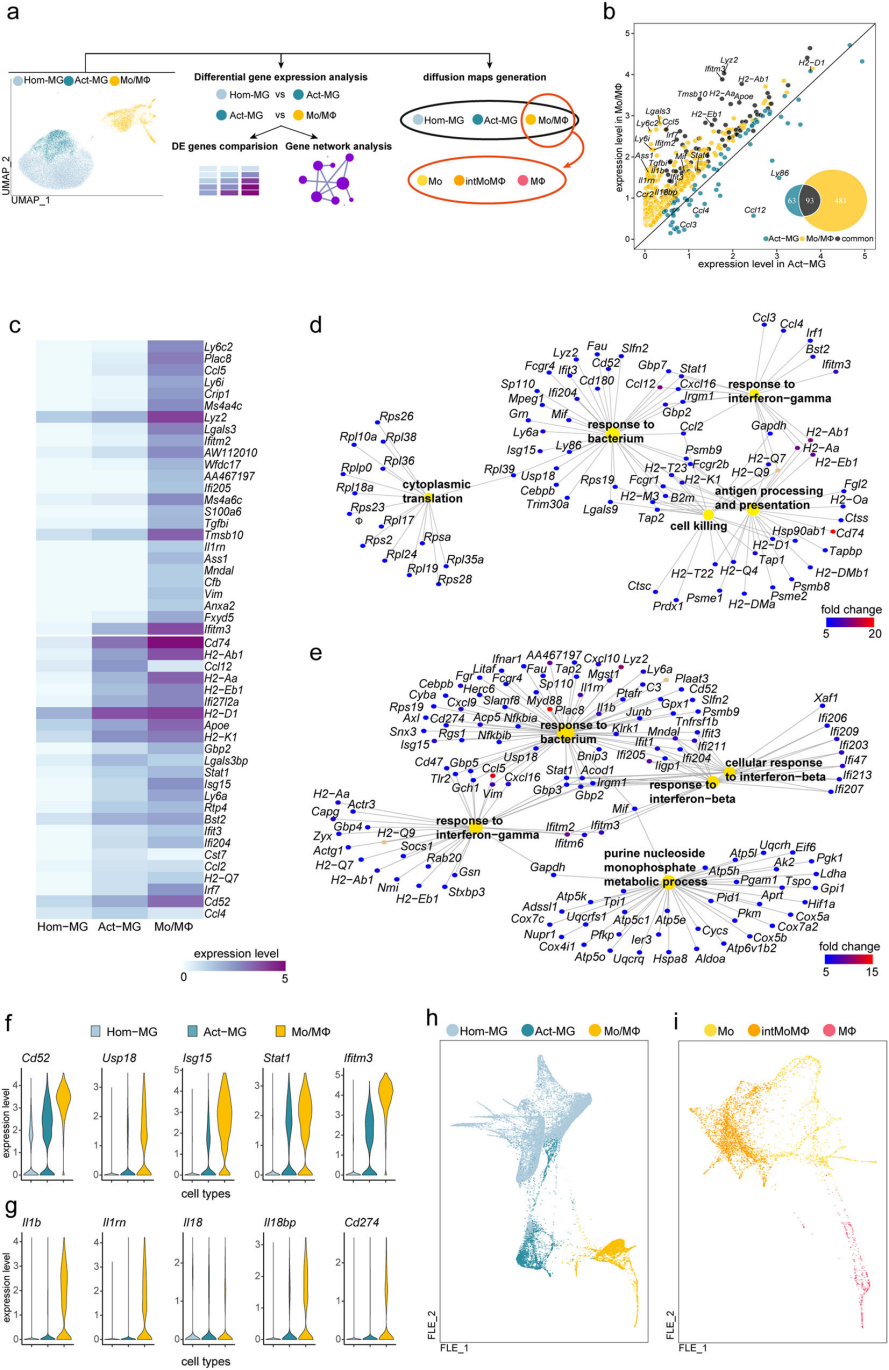
Functional analysis of glioma-activated microglia in comparison to tumor-infiltrating monocytes/macrophages. **a** Scheme of the analytical approach. **b** Scatter plot depicting expression levels of differentially upregulated genes in Act-MG and Mo/MΦ. **c** Heatmap showing the comparison of expression of top 25 upregulated genes in Hom-MG vs Act-MG and Act-MG vs Mo/MΦ. **d**, **e** Gene Ontology analysis of biological processes for genes upregulated in **d** Act-MG compared to Hom-MG and **e** Mo/MΦ compared to the Act-MG. **f**, **g** Expression level of selected genes expressed specifically in distinct subpopulations. **h**, **i** Visualization of cells projection on two-dimensional FLE (force-directed layout embedding) space.

As demonstrated above, MG and Mo/MΦ have distinct gene expression profiles. To elucidate their roles in supporting glioma growth, we examined the transcriptional networks activated in MG and Mo/MΦ in TME. Firstly, we extracted genes highly upregulated in microglial cells from glioma-bearing brains (significantly upregulated genes in Act-MG compared to Hom-MG). Subsequently, we compared those profiles in Act-MG and Mo/MΦ cells (Fig. 4a) to find genes either common or specific for each subpopulation. We found that the majority of genes upregulated in the Act-MG are also expressed by Mo/MΦ, and their expression is usually higher in Mo/MΦ than Act-MG (Fig. 4b, c). Among commonly induced genes, we found *Ifitm3* and a group of genes encoding MHCII proteins (*H2-Aa*, *H2-*Ab1, *H2-D1*, and *H2-K1*). Expression of *Ifitm3* has been reported to demarcate MΦ from MG^10^. We demonstrate that *Ifitm3* is highly expressed in Mo/MΦ, but also in Act-MG (Fig. 4b, c). Act-MG showed a high expression of *Ccl3*, *Ccl4*, and *Ccl12* (chemokine-encoding genes) when compared to Mo/MΦ. In contrast, Mo/MΦ were characterized by high expression of *Ifitm2* and *Ccl5* genes (Fig. 4b, c).

Next, we performed Gene Ontology (GO) analysis of biological processes on two sets of genes—genes significantly upregulated in Act-MG compared to Hom-MG (Fig. 4d), and genes significantly upregulated in Mo/MΦ compared to the Act-MG (Fig. 4e). Gene expression in Act-MG was enriched in terms “cytoplasmic translation”, whereas terms “purine monophosphate metabolic process” were enriched in Mo/MΦ. All other terms were directly related to the immune function and largely shared between upregulated genes in MG and Mo/MΦ. Both populations showed induction of genes related to “response to bacterium” and “response to interferon gamma”; however, those terms encompassed the broader number of genes for Mo/MΦ. In addition, Mo/MΦ demonstrated the enrichment of “response to interferon-beta” genes. The genes coding for MHCII components (e.g., *H2-Aa*, *H2-Ab1*, and *H2-Eb1*) are upregulated in both Act-MG and Mo/MΦ; however, in Act-MG, these genes are represented under “antigen processing and presentation” and “response to interferon gamma” terms, and in Mo/MΦ they are represented only under “response to interferon gamma” term.

Several shared genes (*Cd52, Stat1, Isg15*, *and Usp18)* were expressed at a higher level in Mo/MΦ compared to their levels in Act-MG (Fig. 4f). Proteins encoded by those genes are involved in immune responses: CD52 mediates co-stimulatory signals for T-cell activation and proliferation^49^; Stat1 is a mediator of interferon signaling; Isg15 stabilizes Stat1 preventing premature termination of an inflammatory response^50^; Usp18 negatively regulates *Stat1* expression and termination of interferon-induced genes^51^. Such expression patterns may indicate that both MG and Mo/MΦ initiate some elements of the immune response, with more prominent activation in Mo/MΦ. Among genes that were highly expressed in Mo/MΦ, we found *Il1b* coding for an inflammatory cytokine IL-1β along with *Il1rn* and *Il18b* coding for the inhibitors of pro-inflammatory cytokines (Fig. 4g). These data, together with the high expression of *Cd274* coding for PD-L1 in Mo/MΦ, suggest stronger activation of immunosuppressive pathways in Mo/MΦ (Fig. 4g).

In addition, we used scSVA tool^52^ to generate single-cell diffusion maps and to obtain visualization of our data set on force-directed layout embedding (FLE; Fig. 4h, i). Using previously assigned cell identity labels, we demonstrate with a different computational approach, how cells from each cluster are projected onto two-dimensional space. Analysis showed similar patterns of cell distribution for Hom-MG, Act-MG, and Mo/MΦ cells on FLE (Fig. 4h) as on UMAP (Fig. 3a), as well as for subpopulations of Mo/MΦ cells (Figs. 4i and 2j, respectively). Expression profiles of Mo and MΦ suggest to some extent that transition from Mo to intMoMΦ to differentiated macrophages takes place in glioma microenvironment.

### Sex-related differences in microglial expression of MHCII genes

**Fig. 5:**
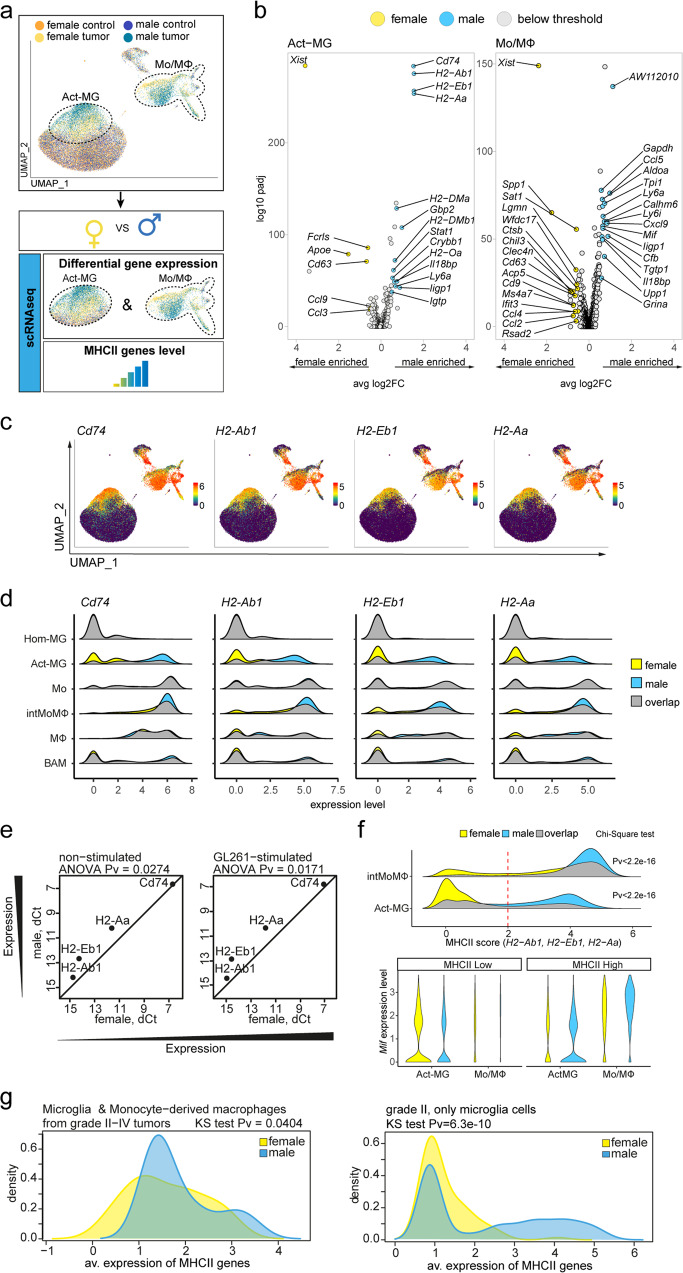
Expression *of* MHCII and *Cd74* genes is more abundant in microglia and monocytes/macrophages from gliomas in males. **a** Illustration of the analytical approach. UMAP plot demonstrates the distribution of male and female cells across cell clusters and reveals sex-enriched areas in Act-MG and Mo/MΦ. Differential gene expression analysis was performed for male vs female in Act-MG and Mo/MΦ groups, and expression level of top differentially expressed genes (DEG) verified across all cell groups. **b** Volcano plots depicting DEG across sexes in Act-MG and Mo/MΦ-infiltrating gliomas. **c** Expression of the most highly upregulated genes from males. **d** Density plots show enrichment of male cells in MHCII genes- and *Cd74*-high expressing populations of Act-MG and intMoMΦ. **e** Gene expression analysis of MHCII and *Cd74* genes in murine primary microglia cocultured with GL261 cells. Gene expression differences determined by qPCR are depicted as dCt, with *Actb* as a housekeeping gene. **f** Violin and density plots demonstrate that *Mif* upregulation is limited to the intMoMΦ MHCII^hi^ cells; two-sided Chi-Square test. **g** Distribution of MHCII genes average expression in human data sets. Left panel shows distribution of the MHCII genes level in microglia and monocyte-derived macrophage cells sorted from glioma grades II–IV samples (males *n* = 5, females *n* = 3)^55^, and right panel in single microglial cells from WHO grade II glioma samples (males *n* = 5, females *n* = 2 (refs. ^10,56^). Difference between the distribution was assessed with Kolmogorov–Smirnov test.

Sex is an important prognostic marker in GBM patients influencing incidence and disease outcomes^11^. Differences between male and female MG in naïve mice have been reported^13,14^. We examined whether there are sex-related differences in gene expression in main myeloid populations in gliomas. The unsupervised cell clustering showed that microglia from glioma-bearing brains, but not from naïve brains, segregate into clusters that are enriched either in cells originating from female or male (Fig. 5a and Supplementary Fig. 10a). Similarly, we observed the sex-driven cell grouping within the intMoMΦ subpopulation, pointing to differences in immune cell activation in male and female mice (Fig. 5a and Supplementary Fig. 10b).

In Act-MG from males, among most highly upregulated genes are *H2-Ab1*, *H2-Eb1*, and *H2-Aa* coding for the components of MHCII and *Cd74*—encoding an invariant MHCII chain implicated in folding and trafficking of the MHCII proteins (Fig. 5b). The increased expression of the MHCII genes and *Cd74* was found in the male-dominated cell clusters in the Act-MG, but also in the intMoMΦ (Fig. 5c). Accordingly, the cells with the high expression of MHCII and *Cd74* genes were enriched in Act-MG and intMoMΦ in males. This enrichment was not observed in Hom-MG, Mo, MΦ, and BAMs (Fig. 5d).

We verified the difference in *Cd74* and MHCII genes expression in another model: male- and female-derived murine primary microglial cells cocultured with GL261 cells (Fig. 5e). Expression levels of the selected genes were sex-dependent with higher expression in male- compared to female-derived cultures. The non-stimulated microglia derived from males also showed increased expression of MHCII genes, which might suggest an intrinsic capability of male microglia to overexpress MHCII genes that is detected already in immature microglia.

IntMoMΦ in males upregulated *Mif* encoding a macrophage migration inhibitory factor. MIF stimulates CCL2-mediated macrophage migration and cell proliferation^53^, and in glioma TME it suppresses antitumoral microglia activity via activation of CD74 (ref. ^54^). The increased *Mif* expression in cells with high expression of MHCII genes was restricted to intMoMΦ and not detected in the Act-MG (Fig. 5f). Although high expression of MHCII genes was found in Act-MG and intMoMΦ from males, its implications in different subpopulations may differ in gliomas.

In addition to the two independent animal and in vitro models, we observed the similar differences in expression of MHCII genes in human samples. We performed an analysis of the MHCII gene expression using bulk RNA-seq on MG and BMDMs derived from WHO grades II–IV glioma patients^55^ (Fig. 5g, left panel) and human data sets obtained in single-cell studies on MG from WHO grade II glioma patients^10,56^ (Fig. 5g, right panel). In line with our findings on mouse gliomas, an average expression of the MHCII genes was higher in males (Fig. 5g). Female samples were underrepresented in this analysis, despite including all data sets from single-cell studies on human gliomas, where sex information was provided. In addition, we tested the human glioma expression data from The Cancer Genome Atlas (TCGA) to determine if sex has an impact on the expression of MHCII and *CD74* genes. GBM samples were not discriminated by expression of the selected MHCII and *CD74* genes (data not shown), irrespective of *IDH1* mutation status or MΦ content as estimated with the xCell^57^. However, the expression of MHCII and *CD74* genes stratified WHO grade II diffuse glioma patients into the female-enriched MHCII^low^ and the male-enriched MHCII^high^ groups (Supplementary Fig. 11). This observation shows that the differential regulation of genes coding for MHCII complex between sexes is not limited to a mouse glioma model, and those differences could be of clinical relevance.

## Discussion

In the present study, we have used flow cytometry and scRNA-seq to dissect the cellular and functional heterogeneity of GAMs. scRNA-seq of CD11b^+^ cells from naïve brains revealed considerable MG heterogeneity suggesting the existence of various functional states of MG in the brain. Main clusters expressed homeostatic and signature genes (MG1)^31^, immediate early genes typical for transcriptionally active cells (MG2), or genes coding for signaling inhibitors and transcriptional repressors (MG3)^22,33^. PreMG cluster was enriched in the “microglia signature”, cytokines, chemokines, and interferon response genes. Similarly, recent work of Sankowski et al.^58^ distinguished several microglial phenotypes within healthy and diseased (glioma and brain metastases) human brains, including a homeostatic subpopulation upregulating core microglia genes, a subpopulation expressing high levels of cytokine genes, and a subpopulation enriched in MHCII genes expressing cells^58^. However, the study did not discriminate peripherally derived MΦ among GAMs, which could interfere with the characterization of the microglial subsets.

MG and BMDMs accumulate in human GBMs and support glioma progression by augmenting tumor invasion, angiogenesis, and inducing immunosuppression^2^. Our main goal was to identify markers and functions of distinct myeloid subpopulations in murine malignant gliomas. Identifying specific roles of various subpopulations is critical for a cell population-specific intervention. Transcriptomic analyses of bulk CD11b^+^ infiltrates from human GBMs and murine gliomas showed a mixture of profiles characteristic for both pro- and antitumor phenotypes^4–6^. Cell separation based on CD45 expression has been criticized as CD45 can be upregulated in microglia under pathological conditions^59^. Herein, we show that *Tmem119* is highly expressed by MG (both in control and glioma conditions), and *Lgals*3 (encoding Gal-3) by infiltrating Mo/MΦ at RNA and protein levels. This pattern allows efficient separation of Tmem119^+^ and Gal-3^+^ cells within CD11b^+^ cells with flow cytometry. Interestingly, Tmem119 and Gal-3 separation of CD11b^+^ largely overlapped with CD45^hi/lo^ separation. Staining for Tmem119 and Gal-3 revealed a non-uniform cell distribution within the tumor, with the predominance of Mo/MΦ (Gal-3^+^) in the tumor core, and MG (Tmem119^+^) occupying the tumor periphery. This observation is in agreement with results showing a distinct spatial distribution of microglia and BMDMs in lineage tracing experiments^48^, high-resolution two-photon imaging^60^, and a scRNA-seq study on matched patient-derived samples from the tumor core and periphery^9^.

MG and MΦ transcription regulatory networks adapt to changing environments^61,62^. Single-cell RNA-seq studies on human GBMs suggested that MG and Mo/MΦ diminish their signature of origin, forming a phenotypic continuum and impeding clear separation^8,10^. On the other hand a CyTOF study showed that MG, Mo and MΦ cell fractions could be distinguished in both gliomas and brain metastases^63^. In our study, the unsupervised cell clustering yielded three cell clusters representing MG, Mo/MΦ, and CNS BAMs. When we tested “microglia” and “macrophage” signatures, the “microglia signature” was indeed lower in glioma Act-MG, but its expression was still high and distinguishable from Mo/MΦ. These observations demonstrate that the transcriptional signature of cell origin is retained in glioma TME.

Genetic lineage tracing studies showed BMDM accumulation in GL261 gliomas and transgenic RCAS-PDGF-B-HA gliomas, and the presence of distinct transcriptional networks associated with tumor-mediated education in MG and recruited BMDMs^7,48^. We demonstrate that GL261 gliomas induce similar transcriptional networks in MG and Mo/MΦ, however, the induction is stronger in Mo/MΦ. This could be related to their prevalent localization within the tumor core, in contrast to MG occupying tumor periphery^9,48^. Mo/MΦ express numerous genes related to immunosuppression, whereas, Act-MG show high expression of genes encoding chemokines, acting as chemoattractants from immune cells in mice^64^.

Mo/MΦ are heterogeneous and based on expression profiles we distinguished Mo (*Ly6c2*^hi^*Ccr2*^hi^), intMoMΦ (*Ly6c2*^hi^*Tgfbi*^hi^), and differentiated macrophages (*Ifitm2*^hi^, *S100a6*^hi^, and *S100a11*^hi^) expressing a high level of *Cd274 (*coding for an immune checkpoint inhibitor—PD-L1). This is in line with a single-cell proteome study on human gliomas demonstrating transition of a Mo to a MΦ, as a result of tumor-specific education^63^. The expression profiles of the identified subpopulations together with patterns of cell distribution obtained with UMAP and FLE diffusion maps suggest that Mo arrive as antitumor cells, and undergo differentiation into protumorigenic MΦ in the glioma microenvironment.

We found only a partial overlap with the recently proposed MΦ-specific markers in gliomas^3,7,23^, but the proportion of Mo/MΦ subpopulations may depend on the particular tumor stage. The occurrence of Mo/MΦ expressing an inflammatory *Il1b*, along with *Il1rn* and *Il18b* (coding for the inhibitors of pro-inflammatory cytokines) and *Cd274* is interesting for its clinical relevance, suggesting that pro-invasive and immunoregulatory functions are split between MG and MΦ in the glioma microenvironment, respectively.

Another important finding refers to sex-dependent differences in microglial responses to glioma. We report that male MG and intMoMΦ from the tumor-bearing hemispheres show higher expression of genes coding for MHCII components and *Cd74*. Evaluation of the *Cd74* and MHCII genes by qPCR in primary MG cultures from male and female brains shows higher expression of these genes in male MG in cocultures with GL261 cells and under basal conditions. While we did not detect the sex differences in MHCII genes in Hom-MG by scRNA-seq, another study on bulk CD11b^+^ RNA-seq showed that MG from male naÏve mice express a higher level of MHCI and MHCII genes, and are more reactive to ATP stimulation^13^. The analysis of TCGA and scRNA-seq human glioma data sets showed sex-related differences in MHCII complex and *CD74* genes in WHO grade II diffusive gliomas, where antitumor immunity may influence outcomes. Such differences were not detected in highly immunosuppressed human GBMs. However, higher expression of the MCHII genes was noted in a bulk RNA-seq data set of MG and BMDMs immunosorted from human GBMs^55^. Thus, this issue should be further explored in human studies. Although women are more susceptible to autoimmune diseases, men have a higher risk of death for a majority of malignant cancers^65^. In the immune checkpoint inhibitor therapy of various cancers, males presented better therapeutic outcomes^12^. While the source of sex differences in cancer incidence and outcome remains unknown, antitumor immunity is a plausible candidate.

In sum, MG in a naïve brain are heterogeneous and represent various functional states. Glioma cells attract and polarize MG and peripheral Mo that migrate to CNS. Whereas infiltrating Mo express some inflammatory markers, they likely differentiate into immunosuppressive MΦ within the tumor. Those cells retain their cell signatures, occupy different tumor niches, and display various degrees of glioma-induced activation and specific functions. Interestingly, we found the stronger upregulation of genes of the MHCII complex in MG and a fraction of Mo/MΦ in male than female mice. Further studies on glioma immunopathology should explore this issue, ensure proper representation of both sexes and avoid extending findings from single-sex studies to the general population.

## Methods

### Animals

Ten-week-old male and female C57BL/6 mice were purchased from the Medical University of Bialystok, Poland. Animals were kept in individually ventilated cages, with free access to food and water, at the temperature of 21–23 °C, 50–60% humidity, under a 12 h/12 h day and night cycle. All experimental procedures on animals were approved by the First Local Ethics Committee for Animal Experimentation in Warsaw (approval no 563/2018 and 764/2018).

### Glioma cell lines

GL261 glioma cells obtained from Prof. Helmut Kettenman (MDC, Berlin, Germany), were cultured in Dulbecco modified essential medium (DMEM) supplemented with 10% fetal bovine serum (FBS, Gibco, MD, USA) and antibiotics (100 U/mL penicillin and 100 µg/mL streptomycin) in a humidified atmosphere of CO_2_/air (5%/95%) at 37 °C (Heraeus, Hanau, Germany).

GL261 luc^+^/tdT^+^ cell line was developed from the GL261 cells. GL261 cells were seeded in antibiotic-free medium and after 24 h transfected with pcDNA3.1(+)/Luc2-tdT plasmid (Addgene) linearized with Notl restriction enzyme (ThermoFisher) using Lipofectamine2000 (ThermoFisher). Cells were maintained in a medium supplemented with 400 µg/mL G-418 Solution (Roche) until complete death of mock-transfected cells. tdT-positive cells were enriched by fluorescence-activated cell sorting, expanded and cryopreserved in FBS with 10% dimethyl sulfoxide (Sigma-Aldrich).

### Implantation of GL261 luc^+^/tdT^+^ glioma cells

Mice (12-week-old) were kept under deep anesthesia with 2% isoflurane during surgery. Using a stereotactic apparatus, a single-cell suspension of GL261 luc^+^tdT^+^ cells (80 000 cells in 1 μL of DMEM) was implanted into the right striatum (+1 mm AP, −1.5 mm ML, and −3 mm DV) at the rate of 0.25 μL per minute. In sham-operated animals, 1 μL of DMEM was injected. To confirm the presence of the tumor, 2 weeks after implantation, animals received an intraperitoneal injection of 150 mg luciferin/kg body weight 10 min prior to imaging with the Xtreme in vivo bioluminescence imaging system (Bruker, Germany). The images were acquired at medium binning with an exposure time of 2 min. X-ray images were acquired at the same mice position with the Xtreme equipment. The signal intensity of the region of interest was computed using the provided software.

### Tissue dissociation

Two weeks after tumor implantation, mice with gliomas, naïve or sham-operated animals (controls) were perfused transcardially with cold phosphate-buffered saline (PBS) to clear away blood cells from the brain. Further processing was performed on the pooled tissue from two animals per replicate. The tumor-bearing hemispheres and corresponding hemispheres from naive animals were dissociated enzymatically to obtain a single-cell suspension with a Neural Tissue Dissociation Kit with papain (Miltenyi Biotec) or 0.5 mg/mL DNase I (DN25, Sigma-Aldrich) in DMEM (Gibco, Germany) with 10% FBS for Tmem119 preparations and gentleMACS Octo Dissociator (Miltenyi Biotec), according to the manufacturer’s protocol. Next, the enzymatic reaction was stopped by the addition of Hank’s Balanced Salt Solution with calcium and magnesium (Gibco, Germany). The resulting cell suspension was filtered through a 70 and 40 μm strainer, and centrifuged at 300 × *g*, 4 °C for 10 min. Next, myelin was removed by centrifugation on 22% Percoll gradient. Briefly, cells were suspended in 25 mL Percoll solution (18.9 mL gradient buffer containing 5.65 mM NaH_2_PO_4_H_2_O, 20 mM Na_2_HPO_4_2(H_2_O), 135 mM NaCl, 5 mM KCl, 10 mM glucose, 7.4 pH; 5.5 mL Percoll (GE Healthcare, Germany); 0.6 mL 1.5 M NaCl), overlayered with 5 mL PBS and centrifuged for 20 min at 950 × *g* and 4 °C, without acceleration and brakes. Next, cells were collected, washed with Stain Buffer (BD Pharmingen), quantified using an EVE™ Automatic Cell Counter (NanoEnTek Inc., USA), and split for CD11b^+^ FACS and cytometric analysis.

### Flow cytometry

Samples were constantly handled on ice or at 4 **°**C avoiding direct light exposure. First, samples were incubated with Live/Dead Fixable Violet Dead Cell Stain (ThermoFisher) or Fixable Viability Dye eF506 (eBioscience) in PBS for 10 min to exclude nonviable cells. Next, samples were incubated for 10 min with rat anti-mouse CD16/CD32 Fc Block™ (BD Pharmingen) in Stain Buffer (BD Pharmingen) to block FcγRIII/II and reduce unspecific antibody binding. Then, cell suspensions were incubated for 30 min with an antibody cocktail in Stain Buffer (BD Pharmingen). For cell surface antigens the following anti-mouse antibodies were used: from BD Pharmigen: CD45 (30-F11), CD11b (M1/70), and Ly6C (AL-21); from BioLegend: CD49d (R1-2); from ThermoFisher: PD-L1 (MIH5); and from Abcam: Tmem119 (106-6). For intracellular staining, Foxp3/Transcription Factor Staining Buffer Set was used (eBioscence), following manufacturer’s instructions. For intracellular antigens, anti-mouse Galectin-3 (M3/38 BioLegend) was used.

Antibodies were titrated prior to staining to establish the amount yielding the best stain index. Samples were acquired using a BD LSR Fortessa Analyzer cytometer. Data were analyzed with FlowJo software (v. 10.5.3, FlowJo LLC, BD). Gates were set based on FMO (fluorescence minus one) controls and back-gating analysis. Percentages on cytograms were given as the percentage of a parental gate. All flow cytometry experiments were performed at the Laboratory of Cytometry, Nencki Institute of Experimental Biology. For reagent specifications, catalog numbers and dilutions see the Supplementary Table 4.

### Sorting of CD11b^+^ cells by flow cytometry

Cells were incubated with Live/Dead Fixable Violet Dead Cell Stain (ThermoFisher) in PBS for 10 min to exclude nonviable cells (Supplementary Fig. 1d). Then, cells were suspended in Stain Buffer (BD Pharmingen) at a density of 1 million cells per 100 μL and stained with anti-mouse CD16/CD32 Fc Block™ (BD Pharmigen) for 10 min. Next, anti-mouse CD11b antibody (M1/70, BD Pharmigen) was added and cells were incubated for 20 min at 4 °C, washed with Stain Buffer, and sorted to 20% FBS in PBS.

### Single-cell RNA sequencing

Directly after sorting, cell quantity, and viability of CD11b^+^ cells were measured, and a cell suspension volume equivalent to 5000 target cells was used for further processing. Preparation of gel beads in emulsion and libraries were performed with Chromium Controller and Single-Cell Gene Expression v2 Chemistry (or v3 for naive vs sham-implanted experiment—Supplementary Fig. 1; 10× Genomics), according to the Chromium Single-Cell 3′ Reagent Kits v2 (or v3) User Guide provided by the manufacturer. Libraries’ quality and quantity were verified with a High-Sensitivity DNA Kit (Agilent Technologies, USA) on a 2100 Bioanalyzer (Agilent Technologies, USA). Next, sequencing was run in the rapid run flow cell and paired-end sequenced (read 1–26 bp, read 2–100 bp) on a HiSeq 1500 (Illumina, San Diego, CA 92122 USA).

### Single-cell RNA-seq data preprocessing and normalization

Raw sequencing data (BCL files) were demultiplexed and converted to fastq files using the CellRanger v3.0.1 (10× Genomics)^66,67^ (https://support.10xgenomics.com/single-cell-gene-expression/software/pipelines/latest/installation) and bcl2fastq v2.20.0.422 (Illumina). Sequencing results were mapped to a mouse genome GRCm38 (mm10) acquired from the 10× Genomics website and quantified, using a CellRanger v.3.0.1 (refs. ^66,67^). The total number of cells identified by the CellRanger was 41,059 (details in Supplementary Table 2). The median number of detected genes per cell was 1059, and the median unique molecular identifiers per cell was 2178. Data analysis was performed in R using Seurat v3 (refs. ^67,68^). Unless otherwise specified in the description, all other quantitative parameters were fixed to default values. To filter out possible empty droplets, low-quality cells, and possible multiplets, cells with <200 or >3000 transcripts were excluded from the analysis. In addition, cells of poor quality, recognized as cells with >5% of their transcripts coming from mitochondrial genes, were excluded from the downstream analysis. After applying these filters, 40,401 cells were present in the data set. Gene expression measurements for each cell were normalized by the total number of transcripts in the cell, multiplied by a default scale factor, and the normalized values were log-transformed (“LogNormalize” method). Following a Seurat workflow, for each replicate the 2000 most highly variable genes were identified, using variance stabilizing transformation (“vst”). To facilitate identification of cell types, these gene sets were expanded by adding genes described as having important roles in immune cells (see Supplementary Table 3) and genes involved in cell cycle regulation^69^. This extension did not influence our conclusions.

### Identification of myeloid cells

Having two biological replicates for each sex and condition (female control, female tumor, male control, and male tumor), data from corresponding samples were integrated using a Seurat v3 approach^67^. To avoid obtaining results fitted too closely to particular data set and therefore possibly failing to fit to additional data, firstly 2000 integration anchors (i.e., cells that are mutual nearest neighbors between replicates) were found. These anchors were then used as an input to the data sets integration procedure. Integrated data were scaled, and unwanted sources of variation, namely total number of counts per cell, percentage of transcripts coming from mitochondrial genes per cell, and cell cycle effect were regressed out, as described in a corresponding vignette [https://satijalab.org/seurat/v3.0/cell_cycle_vignette.html]. Data dimensionality reduction was performed using a principal component analysis, and the first 30 principal components were used in the downstream analyses. For each condition separately, the expression profiles were then clustered using an unsupervised, graph-based approach with the resolution parameter set to 0.3. Clustering results were visualized using two-dimensional *t*-SNE^70^. Based on expression of the reported/canonical markers, the clusters dominated by myeloid cells in four conditions were identified and further analyzed.

### Comparative analysis

The comparative analysis was based on the raw counts, but limited to the previously selected profiles and genes (see above). For such a merged data set, a new set of the 2000 most highly variable genes was identified using variance stabilizing transformation (“vst”), and this set was further expanded by adding the genes involved in cell cycle regulation. Computation of expression estimations, regression of the unwanted variation, and data dimensionality reduction were performed as described above. Next, the expression profiles were clustered using the same approach as above, but with a resolution parameter set to 0.6. After clustering, data were visualized using two-dimensional UMAP^71^. Based on expression of reported/canonical markers of myeloid cells, clusters with cells of interest (microglia, macrophages, and BAMs) were identified. Further analysis of the microglia cluster revealed that some subclusters cells originated in a significant majority from tumor samples. In contrast, there were no subclusters so strongly dominated by cells originated from control samples. Based on that observation, two subsets of microglial cells with distinct transcriptional profiles were identified: Hom-MG and activated microglia (Act-MG).

Differentially upregulated genes (signature genes) were found for each of the identity classes of interest. Significantly upregulated genes between compared groups were found using a Wilcoxon rank-sum test implemented in Seurat v3 (min.pct = 0.25, only.pos = TRUE). These genes were subsequently used for the functional analysis and characterization of the identified clusters. GO analysis was performed using the clusterProfiler v3.12.0 package^72^. In addition, scSVA v0.2 (Docker image version) was used to create FLE plots.

### Human data analysis

MHCII plots were prepared using bulk RNA-seq data from Klemm et al.^55^ (https://joycelab.shinyapps.io/braintime/) and scRNA-seq glioma data from Tirosh et al.^56^^,^^69^ [GSE70630] and Venteicher et al.^10^ [GSE89567]. For scRNA-seq data, only expression profiles from glioma WHO grade II were selected. For each patient, top 10% cells with the highest microglia score were selected. For each of the selected cells an average expression of MHCII complex genes was computed (the list of genes in Table 3). For TCGA data analysis, the normalized expression values for low- and high-grade gliomas were downloaded from TCGA website (RNASeqV2 set available on 07/05/19). Sample annotations and *IDH1* mutation status were obtained from Ceccarelli and colleagues’ study^73^. Content of immune cells was computed with xCell pre-calculated scores downloaded from the xCell website^57^. The genes encoding MHCII and CD74 proteins were selected based on the literature^17,74,75^. The expression profiles were clustered using hierarchical clustering. Significance of the clustering was computed using Fisher’s exact test. Separation of glioma samples was done for each grade separately.

### Immunohistochemistry on brain slices

For tissue collection for histology, mice were anesthetized and transcardially perfused with PBS and 4% paraformaldehyde (PFA). Brains were dissected and fixed in 4% PFA overnight, then placed in 30% sucrose for 2 days, and then embedded in Tissue-Tek O.C.T Compound. Cryosections (10 μm) were cut and stored at −80 °C. Cryosections were blocked in PBS containing 10% donkey serum in 0.1% Triton X-100 solution for 2 h and incubated overnight at 4 °C with rat anti-Gal-3 and rabbit anti-Tmem119 antibodies. Next, sections were washed in PBS and incubated with corresponding secondary antibodies for 2 h at room temperature. Nuclei were counter-stained with DAPI (0.1 mg/mL). Images were obtained on a Leica DM4000B fluorescent microscope. All antibodies were diluted in 0.1% Triton X-100/PBS solution containing 3% of donkey serum. For reagent specifications, catalogue numbers, and concentrations, see Supplementary Table 4.

### Primary microglia and GL261 cocultures

Primary microglial cultures were prepared from cerebral cortices of P0–P2-old-C57BL/6 J male and female mice as described in Walentynowicz et al.^3^. Microglial cells were seeded onto round glass coverslips at 5 × 10^5^ per well in a six-well plate and GL261 cells were seeded onto 0.4 µm inserts (Falcon) at 1.25 × 10^5^ cells per insert. Twenty four hours after seeding, the inserts were transferred into the plate with microglial cells and cocultured for 48 h.

### Quantitative gene expression analysis

RNA was isolated using RNeasy Mini Kit (QIAGEN, USA) and RT-PCR was performed using SuperScript III Reverse Transcriptase (Invitrogen) on 500 ng of total RNA. Quantitative real-time PCR was performed on 40 ng of cDNA in duplicates using TaqMan™ Fast Advanced Master Mix (ThermoFisher) and TaqMan™ Gene Expression Assay (FAM) probes (ThermoFisher). Following probes were used: Cd74 (Mm01262765_g1), H2-Aa (Mm00439211_m1), H2-Ab1 Mm00439216_m1, H2-Eb1 (Mm00439221_m1), Gapdh (Mm99999915_g1), and Actb (Mm00607939_s1). Ct values were normalized to the endogenous expression of Actb and Gapdh. Similar results were obtained for both housekeeping genes, thus only Actb is presented. Delta Ct values obtained for technical replicates (*n* = 2) were averaged and the linear model was built only with biological replicates (*n* = 2). Apart from sex, also litter of animals and genes were used as covariates. The analysis was done with R statistical environment.

### Reporting summary

Further information on experimental design is available in the Nature Research Reporting Summary linked to this paper.

## Supplementary information

Supplementary Information

Description of Additional Supplementary Files

Supplementary Data 1

Reporting Summary

## Data Availability

Data that support findings of this study (bam files and Seurat v3 processed gene expression matrix for each condition) are available in NIH GEO database with the accession number GSE136001.

## Code availability

The code to reproduce the analyses and figures described in this study can be found at: github.com/jakubmie/2019_OchockaSegit.
