# Supplementary material for: Single-cell RNA sequencing reveals functional heterogeneity of glioma-associated brain macrophages
Source: Nat Commun. 2021 Feb 19;12:1151. doi: 10.1038/s41467-021-21407-w (PMC7895824; doi:10.1038/s41467-021-21407-w)
Supplement: Supplementary file 1 — Supplementary Information [file 41467_2021_21407_MOESM1_ESM.pdf]

Supplementary information

Single-cell RNA sequencing reveals functional heterogeneity of glioma-associated brain macrophages

Natalia Ochocka[1#], Pawel Segit[1#], Kacper Adam Walentynowicz[1], Kamil Wojnicki[1], Salwador Cyranowski[1,2], Julian Swatler[3], Jakub Mieczkowski[1*], Bozena Kaminska[1*]

b.kaminska@nencki.edu.pl

j.mieczkowski@nencki.edu.pl

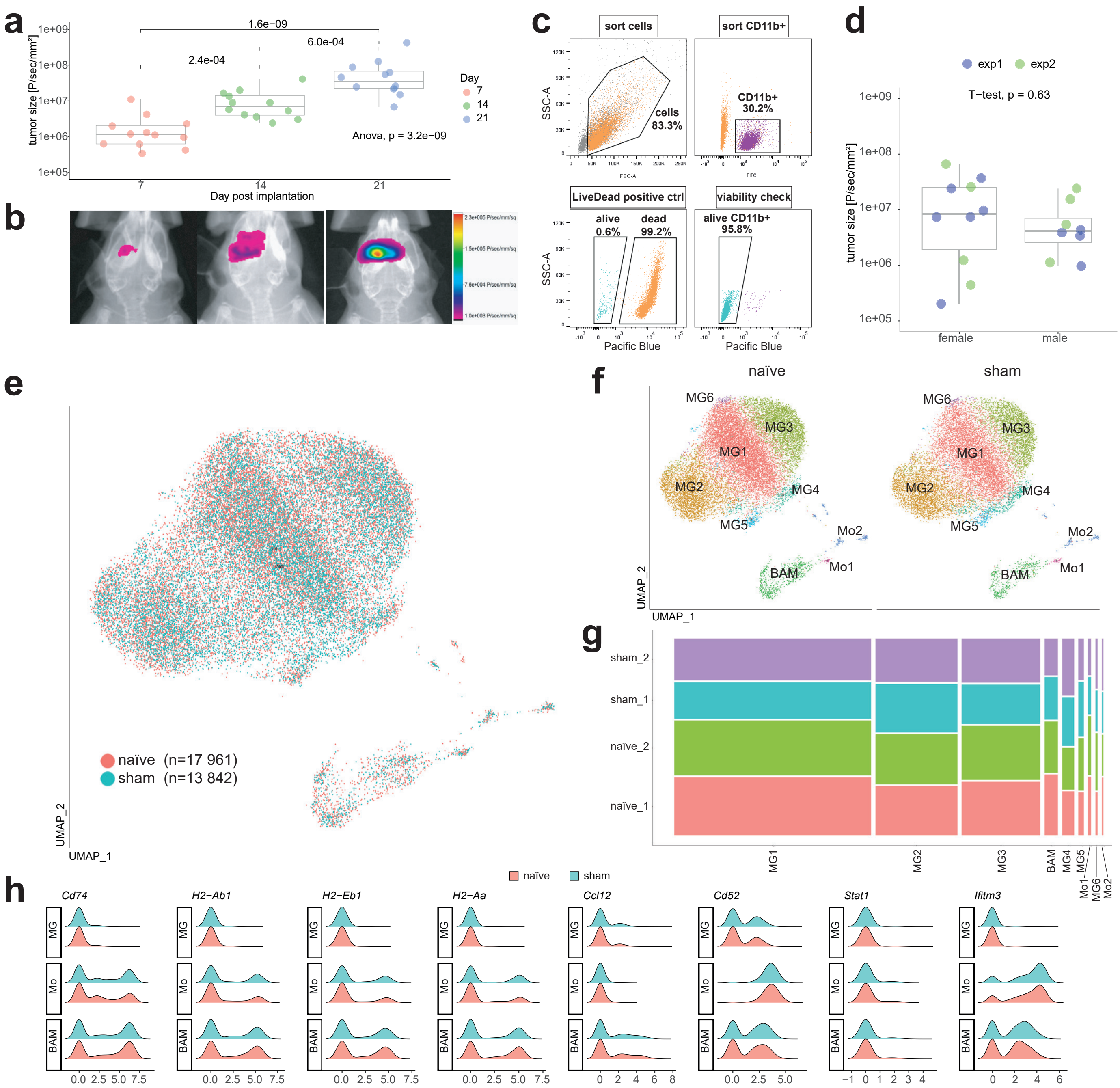

Supplementary Figure 1. Animal model characterization.
(a) Quantification of bioluminescence tumor imaging at 7, 14 and 21 day post-implantation. One-way ANOVA and Tukey's HSD post hoc test, the lower and upper hinges of the boxplots correspond to 25th and 75th percentile, whiskers range from -1.5 IQR to 1.5 IQR and a bar in the center of the box represents a median value, n=12 animals per time point. **(b)** Representative tumor images (tumors for which the bioluminescent signal was closest to the median value in a given time point). **(c)** CD11b+ sorting strategy with labelling for live/dead cell showing high viability of sorted cells, with positive control for dead cells. **(d)** Quantification of bioluminescence tumor imaging male vs female animals for two experiments: exp1- animals sacrificed for scRNA-seq and cytometry measurements, exp2 – animals sacrificed for immunohistochemistry staining. Two-sided t-test, the lower and upper hinges of the boxplots correspond to 25th and 75th percentile, whiskers range from -1.5 IQR to 1.5 IQR and a bar in the center of the box represents median value, n=12 for female and n=10 for male. **(e-h)** Single-cell RNA-seq for CD11b+ cells sorted from brains of naïve and sham-implanted animals. **(e)** UMAP shows uniform distribution of cells from naïve and sham-implanted animals. **(f)** UMAPs demonstrating that all obtained clusters were present for both naïve and sham condition **(g)** Proportion of cells from naïve and sham-implanted animals was comparable across all obtained clusters. **(h)** Expression level of genes showing upregulation in Act-MG compared to Hom-MG (Figure 4c), was not changed in sham-implanted compared to control animals.

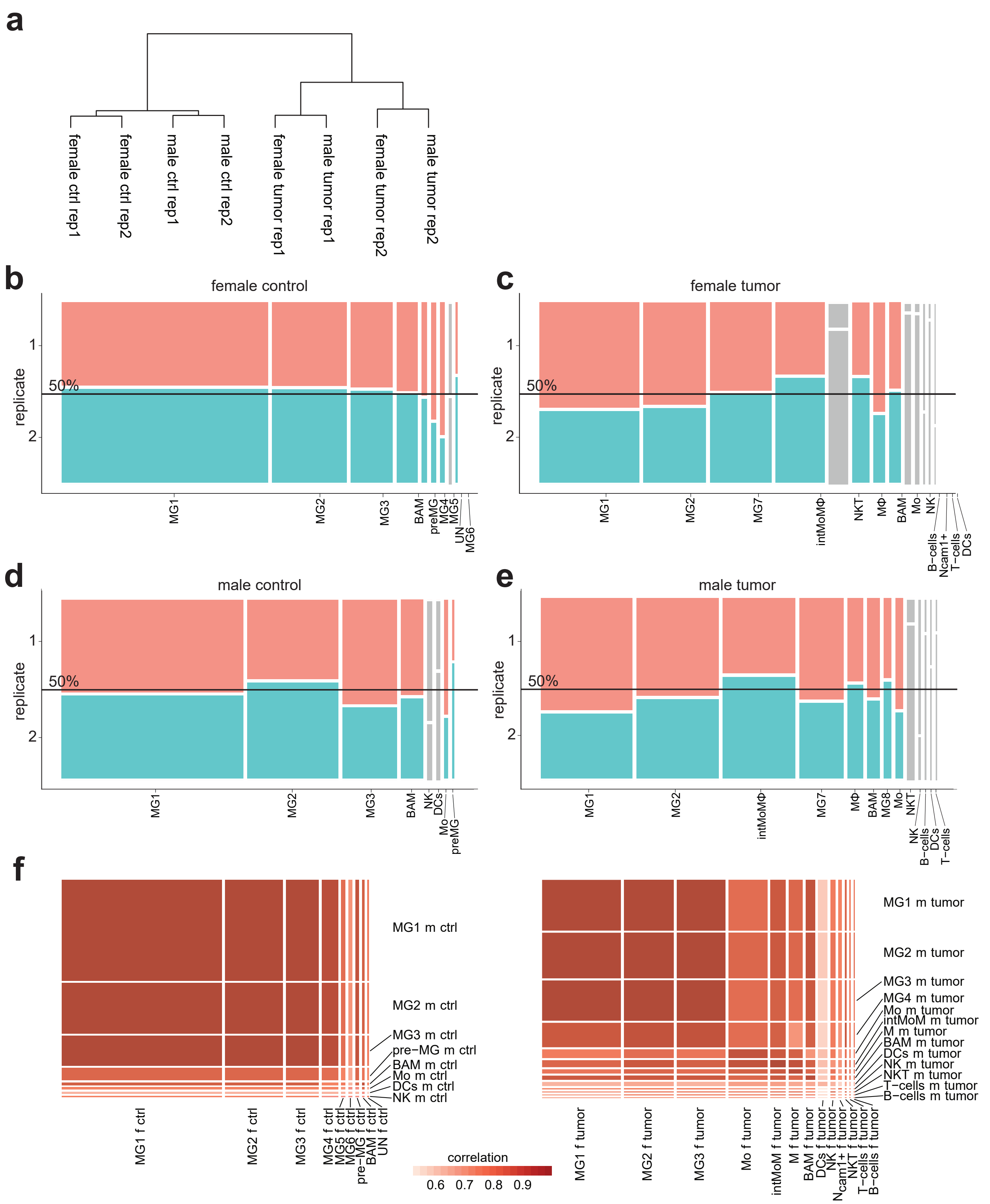

Supplementary Figure 2. Comparsion of the scRNA-seq replicates.
(a) Dendrogram showing results of unsupervised hierarchical clustering of pseudo-bulk gene expression profiles of each sample. **(b-e)** Percentage of cells from 2 replicates in clusters for **(b)** female control, **(c)** female tumor, **(d)** male control, **(e)** male tumor. Width of the each bar corresponds to the size of the cluster. Clusters that were selected for further analysis (Figure 2a) are in red and blue, the other clusters are in grey. **(f)** Correlation heatmaps comparing gene expression profiles of the identified clusters between sexes. Right and Left heatmap correspond to control and tumor samples respectively. Width and height of the cell represents fraction of cells joint into the corresponding cluster.

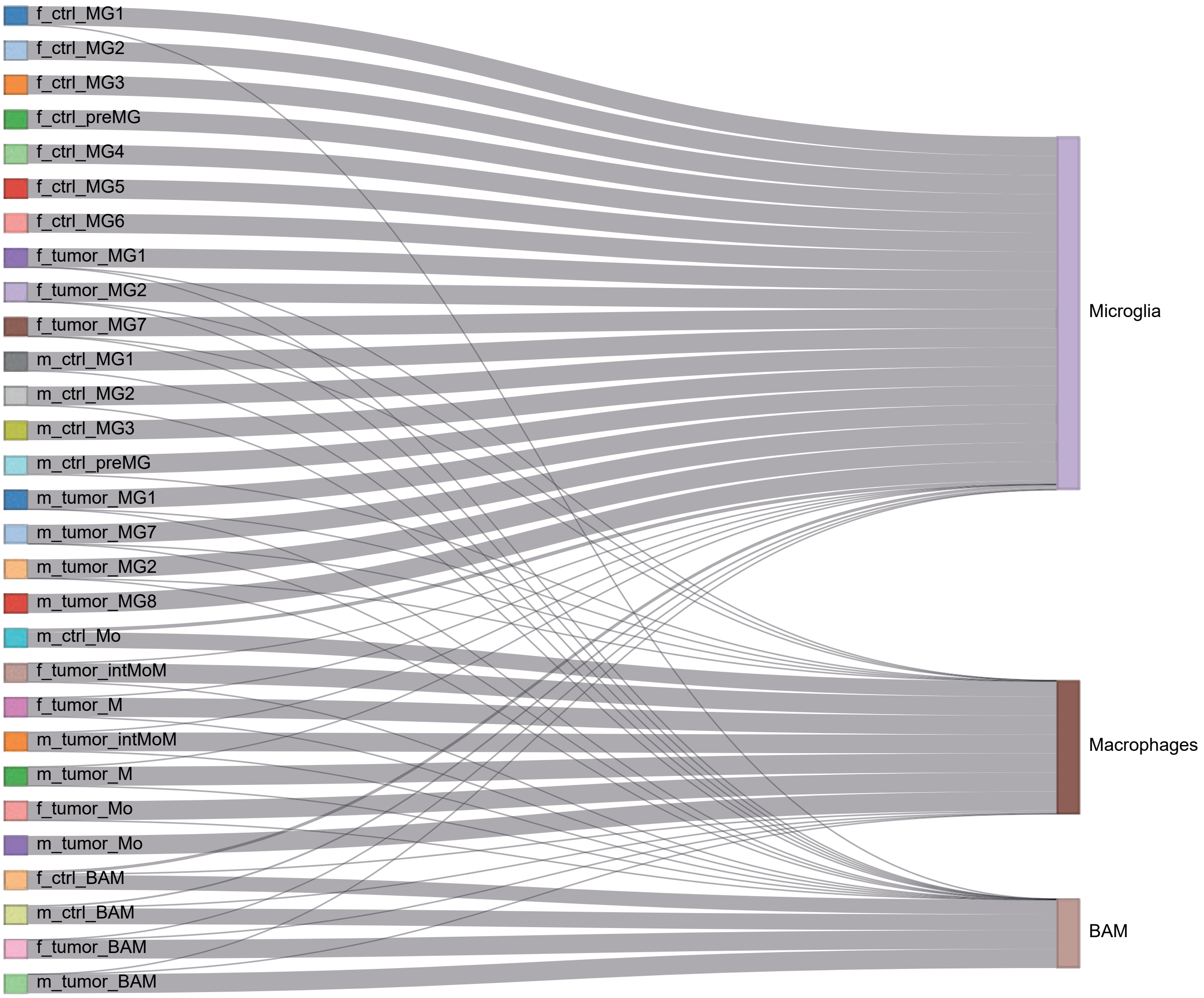

Supplementary Figure 3. Distribution of the identified clusters across Microglia, Macrophages and BAMs clusters. Flow diagram illustrating how cells from clusters obtained in analysis of each condition separately (Figure 1a) have transferred to clusters identified as Microglia, Macrophages, BAMs (Figure 2a), after merging selected cells from all samples into one dataset. Width of each link is proportional to the fraction of cells that have transferred from clusters on the left to clusters on the right side of the plot.

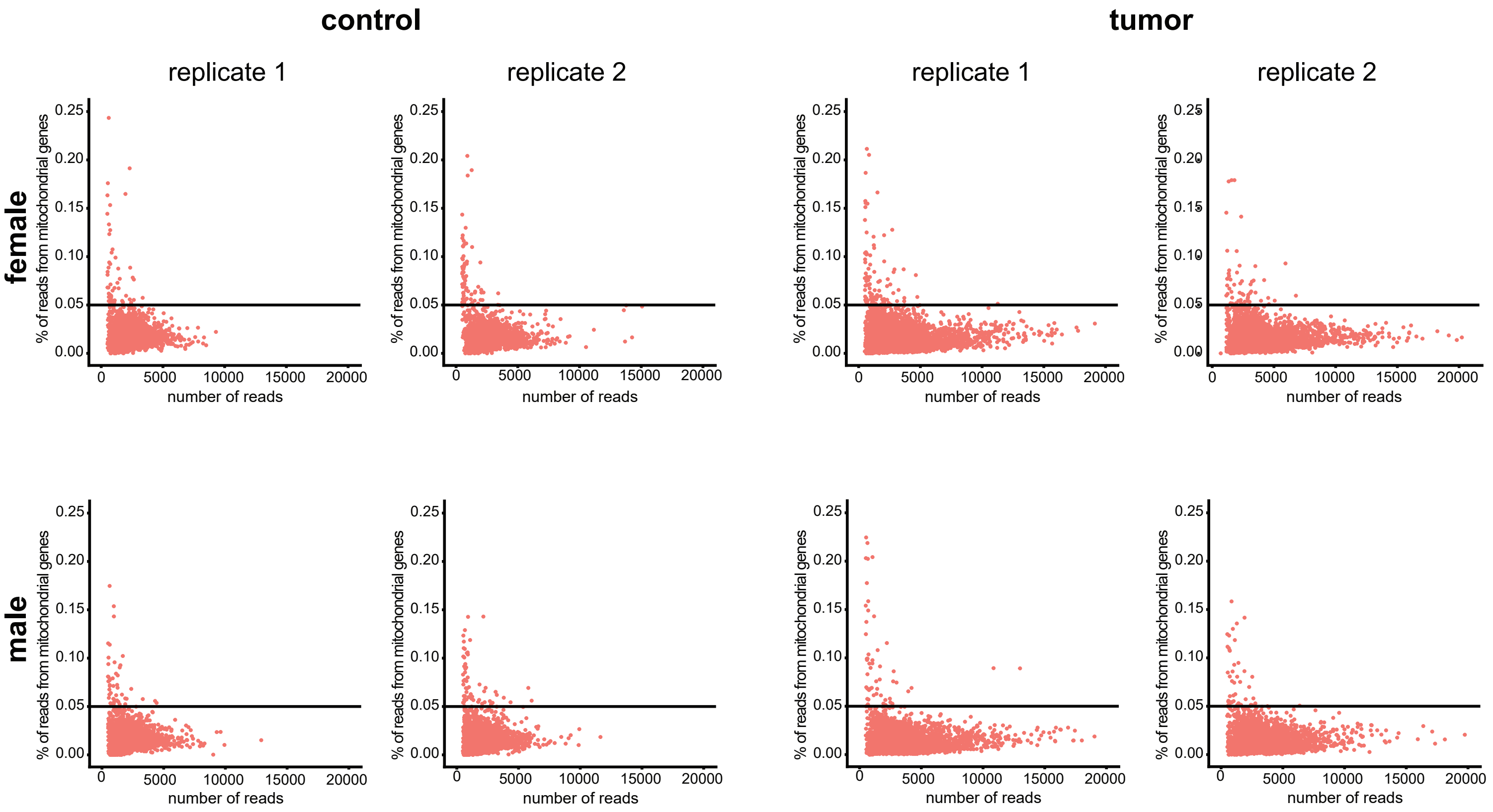

Supplementary Figure 4. Percentage of the mitochondrial reads across samples. Scatter plots visualizing percentage of the reads aligned to mitochondrial genes (Y axis) compared to the total number of reads (X axis). Each dot corresponds to individual cell. The figure is organized as Figure 1b.

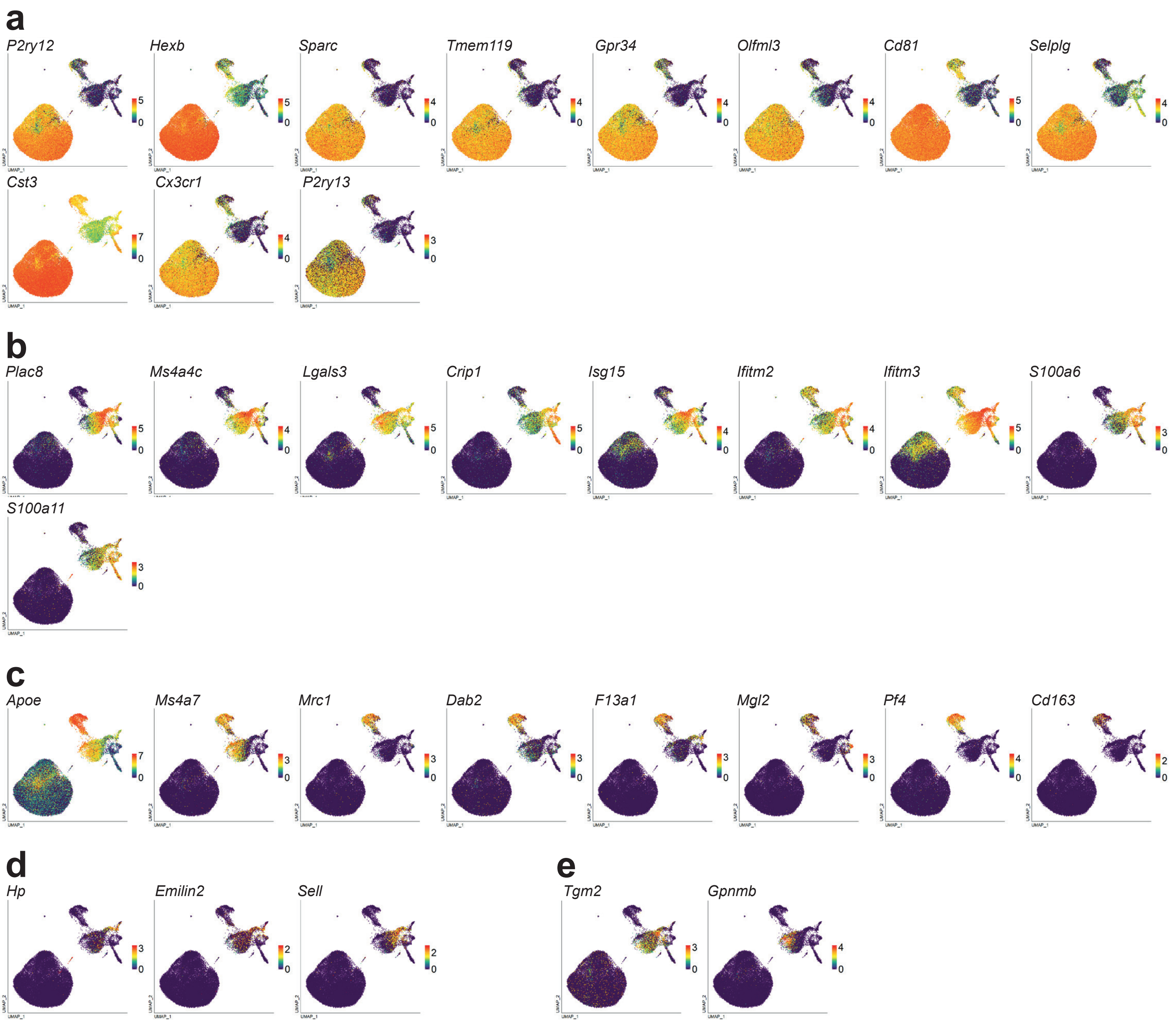

Supplementary Figure 5. UMAP plots demonstrating the distribution of gene expression level for genes **(a)** highly expressed by microglia, **(b)** highly expressed by macrophages, **(c)** highly expressed by CNS Border Associated Macrophages (BAM), **(d)** proposed as markers of monocytes/macrophages infiltrating glioma TME by Haage et al. (2019)[11], **(e)** proposed as markers of Glioma Associated Macrophages (GAM) by Walentynowicz et al. (2018)[10].

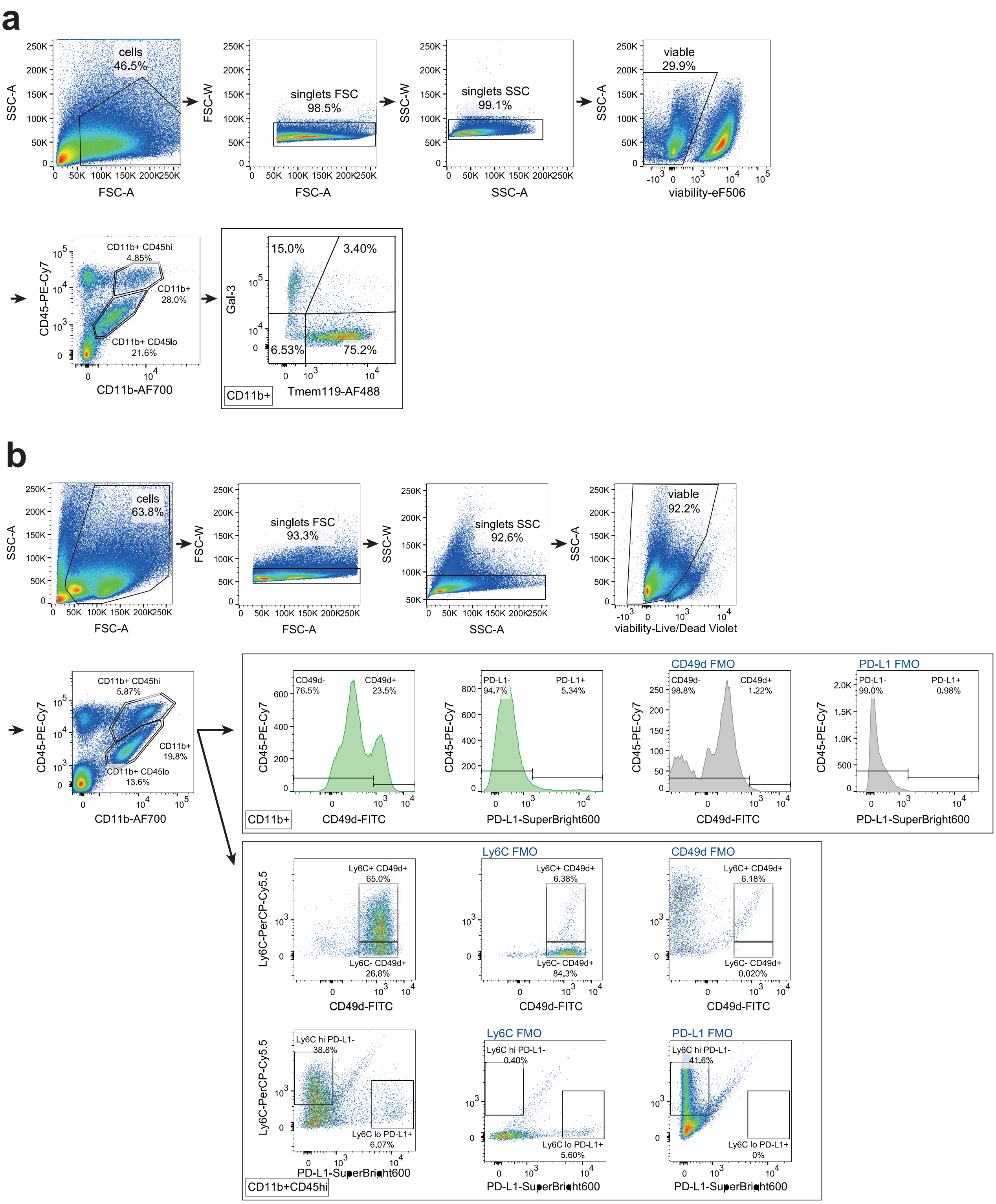

Supplementary Figure 6. Gating strategy for flow cytometry analysis.
(a) Gating for Tmem119 and Gal-3 presented on Figure 2e. **(b)** Gating for CD49d, PD-L1 and Ly6C presented on Figure 2h,i. Events corresponding to cells were gated on SSC-A vs FSC-A plots, then doublets were excluded. Events in singlets gate were further analyzed for the uptake of Fixable Viability Dye (a) or LiveDead Violet dye (b) to exclude events corresponding to dead or damaged cells. For the gating of Tmem119+ and Gal-3+ events (a), gates were set on CD11b+ events. For CD49d+ and PD-L1+ events (b), gates were set on CD11b+ events, and for Ly6C vs CD49d and Ly6C vs PD-L1 analysis (b), gates were set on CD11b+CD45hi events. Gates were set based on backgating strategy or FMO controls. Tissues were dissociated enzymatically with DNase I (a) or papain-based enzyme mix (b) with simultaneous mechanical processing.

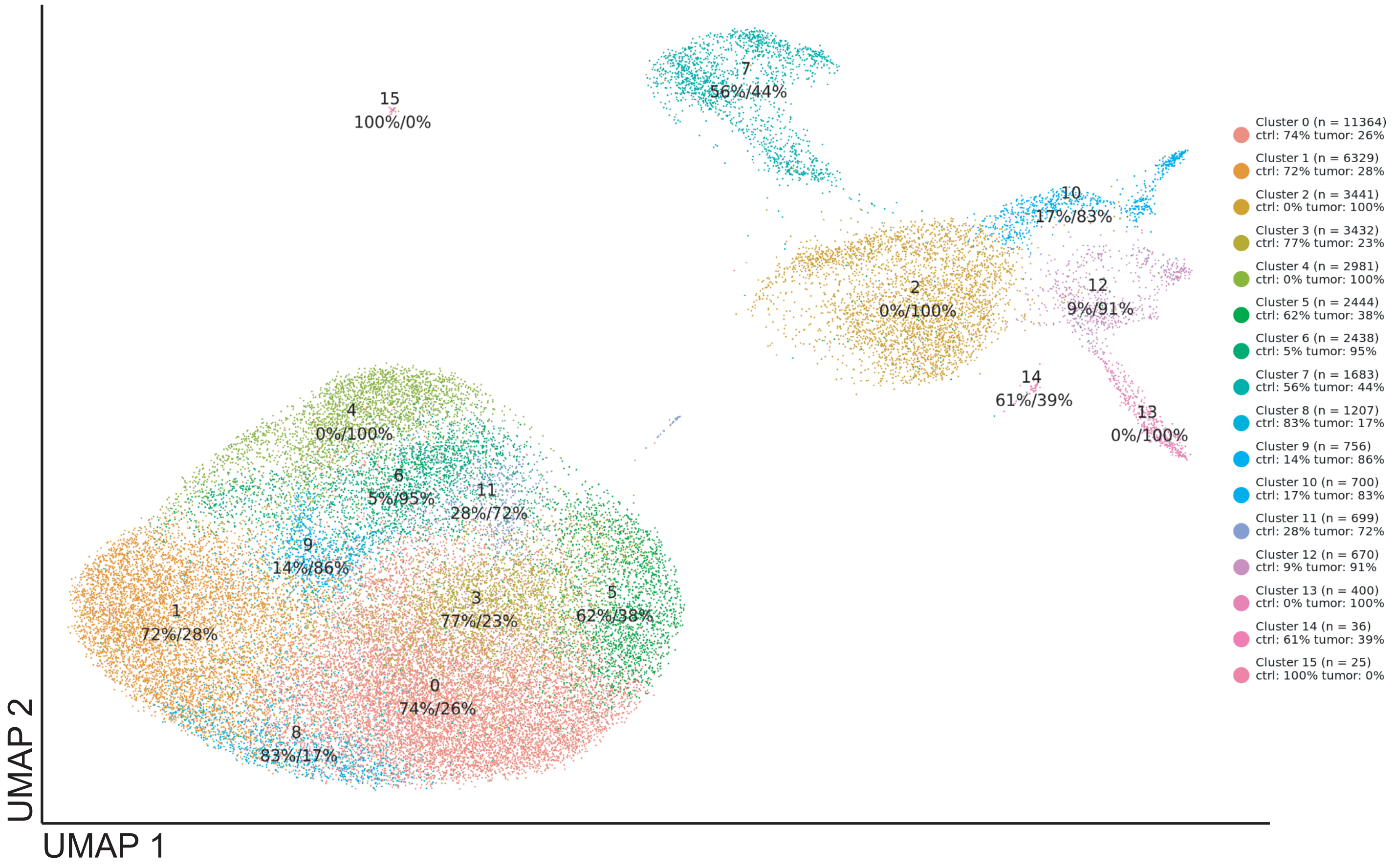

Supplementary Figure 7. Clustering of cells identified as Microglia, Monocytes/Macrophages and BAMs from all conditions.

Results of unsupervised clustering of cells from three subpopulations of interest (corresponding to Figure 2a) from all 8 samples, visualized on UMAP plot. Clusters are marked with different colors and number of cells assigned to each cluster (n) is depicted in the legend. The shown percentages correspond to fractions of cells originating from control and tumor samples respectively.

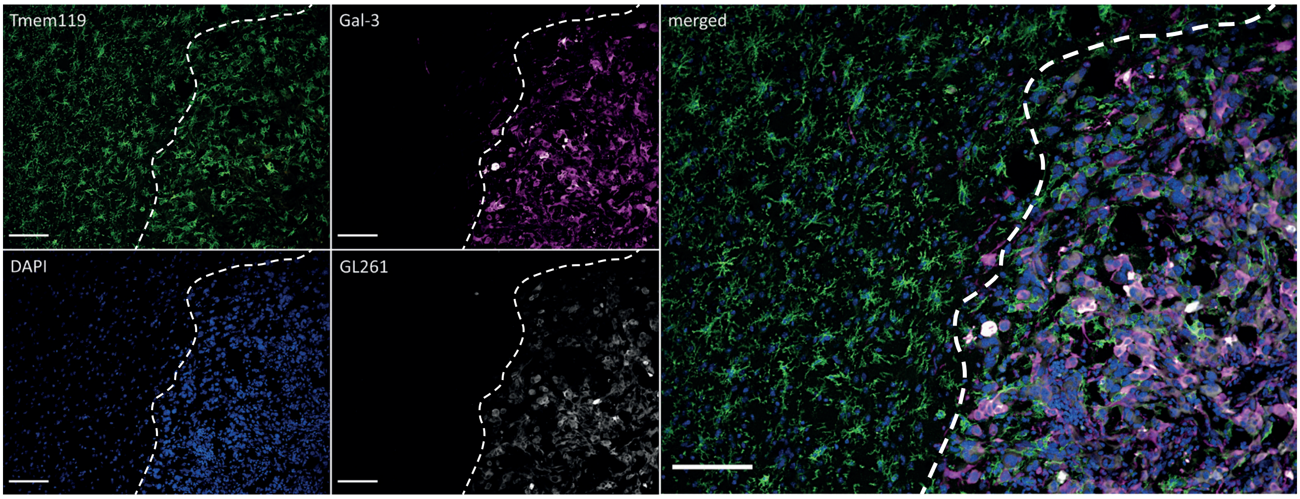

Supplementary Figure 8. Localization of Tmem119+ and Gal-3+ cells in the tumor area
Immunohistochemical staining for microglia (Tmem119+) and Mo/MΦ (Gal-3+) shows the localization of specific immune cells within the tumor and its surroundings in male animal (for female see Figure 3d); a dashed line marks the tumor edge; scale – 100 µm; the staining was performed for 3 animals, 4 sections each, only representative image is shown.

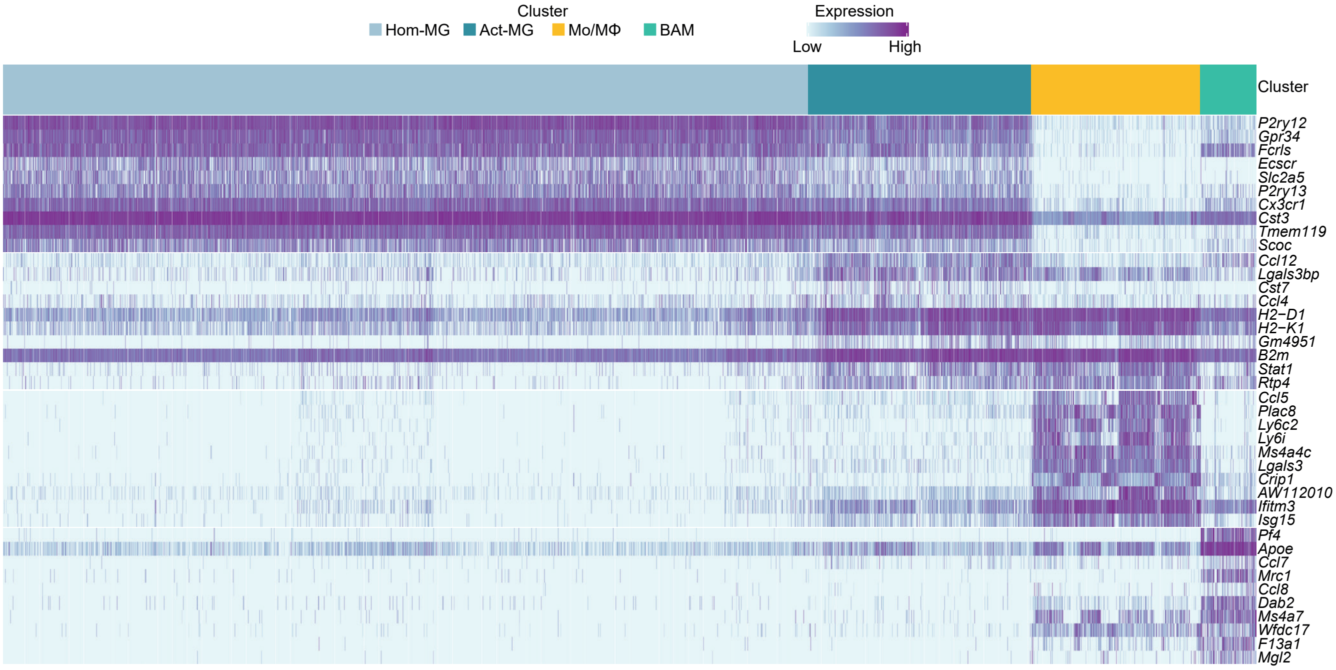

Supplementary Figure 9. Top differentially expressed genes
Heatmap demonstrating top 10 differentially expressed genes in Hom-MG, Act-MG, Mo/MΦ and BAMs (clusters correspodning to Figure 4a).

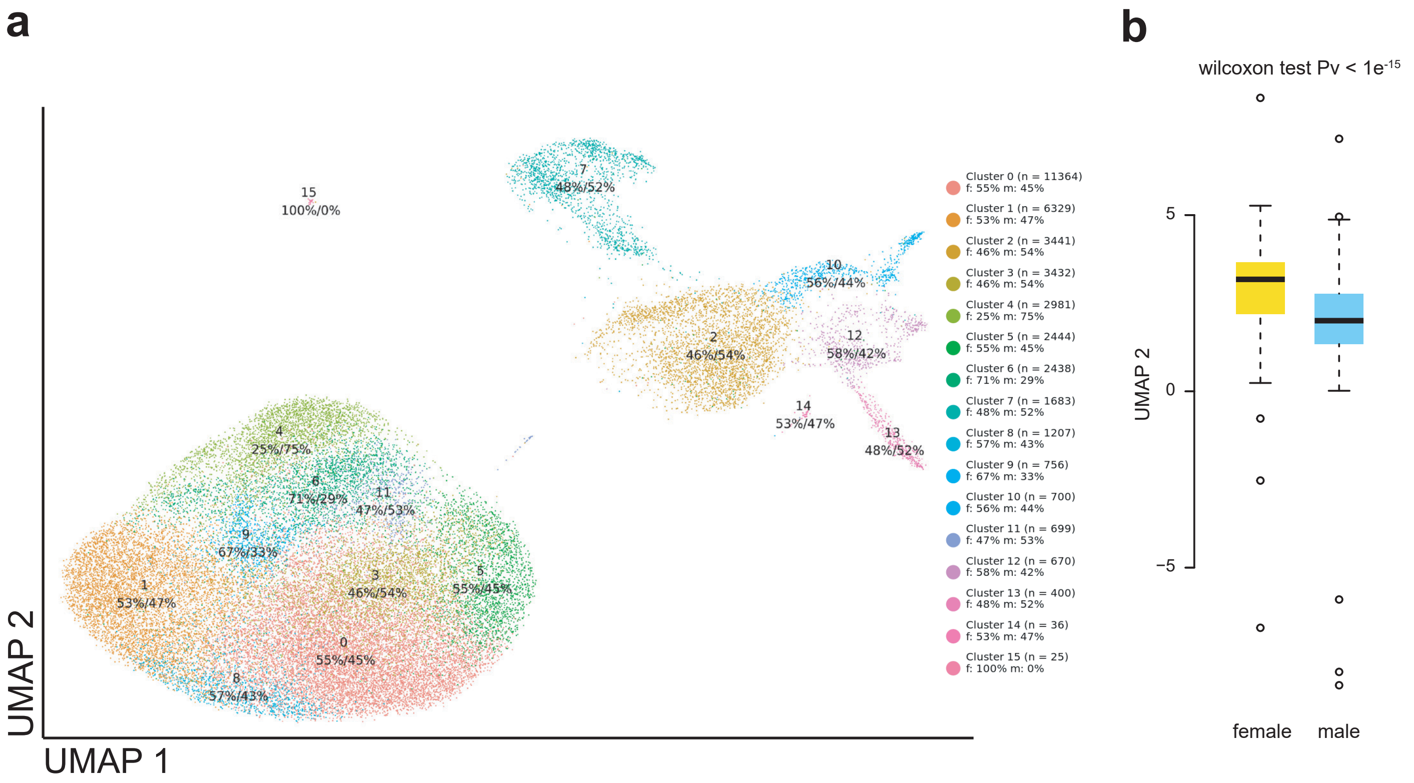

Supplementary Figure 10. Sex-driven cell grouping within the intMoMΦ subpopulation **(a)** Results of unsupervised clustering of cells from three subpopulations of interest (corresponding to Figure 2a) from all 8 samples, visualized on UMAP plot. Clusters are marked with different colors and number of cells assigned to each cluster (n) is depicted in the legend. The shown percentages correspond to fractions of cells originating from female and male samples respectively. **(b)** Comparison of UMAP 2 values for cells originating from female and male samples in cluster #2 identified as intermediate Monocyte-Macrophage cells - intMo/MΦ. Two-sided wilcoxon test, the lower and upper hinges of the boxplots correspond to 25th and 75th percentile, whiskers range from -1.5 IQR to 1.5 IQR and a bar in the center of the box represents a median value, outliers are presented as single points, n=1869 cells for male and n=1572 cells for female.

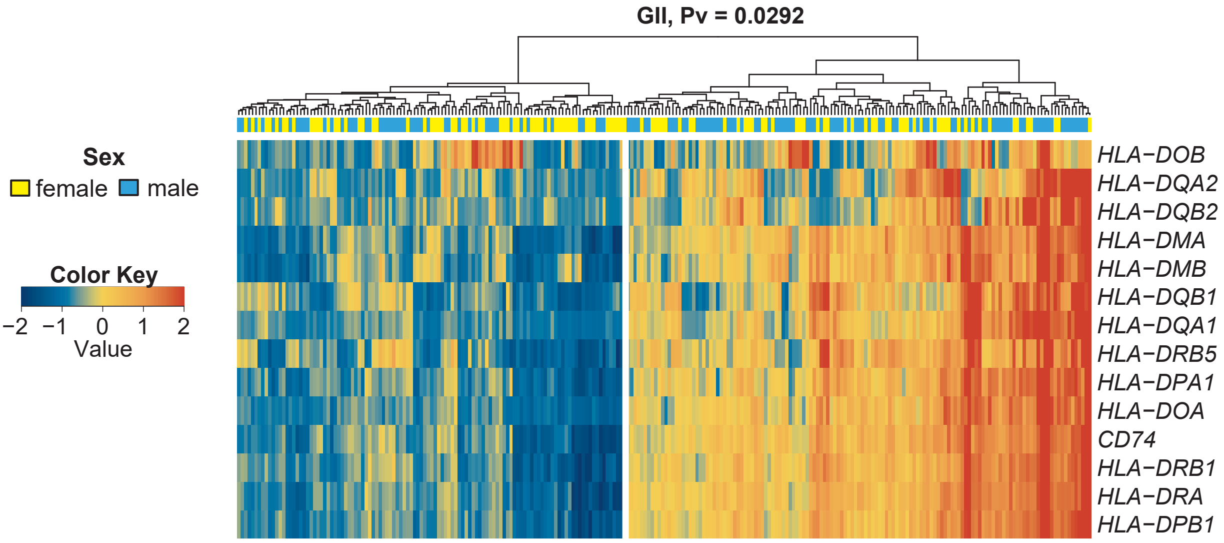

Supplementary Figure 11. *Cd74* and MHCII genes expression across male and female grade II glioma patients

Normalized log2 RNA-seq counts for MHCII complex genes from TCGA WHO grade II glioma patients' data set shows significant differences between male and female glioma patients (two-sided Fisher's exact test).

Supplementary Table 1. List of literature-based markers used to create an immune marker panel for characterizetion of cell identity of obtained clusters (Figure 1b,c).

Gene	Target group	Ref.
Ptprc	hematopoietic cells	1
Itgam	myeloid cells	1
Cd14	myelomonocytic cells	1
Tmem119	microglia	2,3,4
Cx3cr1	microglia	2
P2ry12	microglia	2,3
P2ry13	microglia	2,3
Gpr34	microglia	2,3
Olfml3	microglia	2
Selplg	microglia	2,4
Sparc	microglia	2
Fcrls	microglia	2,3
Siglech	microglia	2
Slc2a5	microglia	2,4
Pf4	microglia progenitors	5
F13a1	microglia progenitors	5
Lyz2	microglia progenitors	5
Ifit3	microglia progenitors	5
Mcm5	early microglia	5
Dab2	early microglia	5
Cxcr2	pre-microglia	5
Scd2	pre-microglia	5
Psat1	pre-microglia	5
Csf1	pre-microglia	5
Crybb1	pre-microglia	5
Fcrls	pre-microglia	5
Selplg	adult microglia	5
Mafb	adult microglia	5
Pmepa1	adult microglia	5
Cd14	adult microglia	5
Lpl	disease associated microglia	6
Cst7	disease associated microglia	4,6
Itga4	macrophages	7,9
Tgfbi	macrophages	8,9
Ifitm2	macrophages	8,9
Ifitm3	macrophages	8
Tagln2	macrophages	8

Gene	Target group	Ref.
F13a1	macrophages	8
Fpr3	macrophages	9
Kynu	macrophages	9
S100a11	macrophages	9
S100a6	macrophages	
Tgm2	Glioma Associated Microglia/Macrophages	10
Gpnmb	Glioma Associated Microglia/Macrophages	10
Emilin2	macrophages in high-grade glioma enviorment	11
Gda	macrophages in high-grade glioma enviorment	11
Hp	macrophages in high-grade glioma enviorment	11
Sell	macrophages in high-grade glioma enviorment	11
Cd163	Border Associated Macrophages	12
Mrc1	Border Associated Macrophages	12,13
Lyve1	Border Associated Macrophages	12
Siglec1	Border Associated Macrophages	12
Ly6c1	monocytes	14
Ly6c2	monocytes	14
Ccr2	classical monocytes	14,15
Spn (CD43)	non-classical monocytes	14
Ly6g	Granulocytes	16
Cd24a	granulocytes/ dendritic cells	16
Itgax	dendritic cells	17
Bst2	plasmocytoid dendritic cells	17
Ncam1	NK cells	18
Klrb1c	NK cells	18
Klrk1	NK cells	18
Ncr1	NK cells	18
Cd2	T-cells, NK cells	1
Cd3d	T cells	1
Cd3e	T cells	1
Cd3g	T cells	1
Cd4	helper T cell	1
Cd8a	cytotoxic T cells	1
Cd8b1	cytotoxic T cells	1
Cd19	B-cells	1
Ms4a1	B-cells	1
Sdc1	B-cells	1

Supplementary Table 2. Number of identified cells, reads per cell and obtained stauration as well as analyzed cells and genes after filtration for each replicate.

	female				male					
	control rep1	control rep2	tumor rep1	tumor rep2	control rep1	control rep2	tumor rep1	tumor rep2	mean	sum
number of identified cells	5 223	4 870	5 802	5 579	4 873	5 301	4 402	5 009	5 150	41 059
number of reads per cell	42 512	33 630	31 190	31 680	35 228	37 195	43 450	31 842	36 412	
saturation	90.1	87.0	83.8	84.7	88.6	89.5	86.2	84.9	87	
number of cells remaining after filtration	5 167	4 787	5 654	5 491	4 820	5 239	4 306	4 937	5 066	40 401
number of genes remaining after filtration	12 520	12 720	13 424	13 192	12 636	12 781	12 978	13 030		

Supplementary Table 3. The list of genes described as having important role in immune cells and involved in cell cycle regulation used to facilitate cell type identification.

Anln	*Cd27*	*Cxcl12*	*H2-Ea-ps*	*Itgb7*	*P2ry13*	*Timp2*
Anp32e	*Cd274*	*Cxcl16*	*H2-Eb1*	*Jun*	*Pcna*	*Tipin*
Anxa1	*Cd34*	*Cxcl2*	*H2-K1*	*Junb*	*Pf4*	*Tlr7*
Anxa5	*Cd38*	*Cxcl9*	*H2-Q7*	*Jund*	*Plac8*	*Tlr9*
Apoe	*Cd3d*	*Cxcr2*	*H2-T23*	*Kif11*	*Pmepa1*	*Tmem119*
Arg1	*Cd3e*	*Cybb*	*H2afv*	*Kif20b*	*Pola1*	*Tmem123*
Ass1	*Cd3g*	*Cycs*	*H2afy*	*Kif23*	*Pold3*	*Tmpo*
Atad2	*Cd4*	*Dab2*	*H2afy2*	*Kif2c*	*Prim1*	*Tnf*
Aurka	*Cd40*	*Dlgap5*	*H2afz*	*Kit*	*Pros1*	*Top2a*
Aurkb	*Cd52*	*Dscc1*	*H3f3a*	*Klf2*	*Psat1*	*Tpx2*
Basp1	*Cd63*	*Dtl*	*H3f3b*	*Klf4*	*Psrc1*	*Trafd1*
Bhlhe41	*Cd72*	*Dusp1*	*Hells*	*Klf6*	*Ptprc*	*Trem2*
Birc5	*Cd74*	*Dusp2*	*Hjurp*	*Klrb1c*	*Rad51*	*Ttk*
Blm	*Cd81*	*Dusp3*	*Hmgb2*	*Klrk1*	*Rad51ap1*	*Tubb4b*
Bmp2k	*Cd8a*	*Dusp5*	*Hmmr*	*Lbr*	*Rangap1*	*Tyms*
Brip1	*Cd8b1*	*Dusp6*	*Hp*	*Lgals3bp*	*Relb*	*Ube2c*
Bst2	*Cd9*	*E2f8*	*Ier5*	*Lpl*	*Rfc2*	*Ubr7*
Bub1	*Cdc20*	*Ecscr*	*Ifi205*	*Ly6c1*	*Rnh1*	*Uhrf1*
Casp8ap2	*Cdc25c*	*Ect2*	*Ifit3*	*Ly6c2*	*Rpa2*	*Ung*
Cbx5	*Cdc45*	*Egr1*	*Ifitm2*	*Ly6g*	*Rrm1*	*Usp1*
Ccl12	*Cdc6*	*Emilin2*	*Ifitm3*	*Ly6i*	*Rrm2*	*Wdr76*
Ccl17	*Cdca2*	*Exo1*	*Il10*	*Lyz1*	*S100a11*	*Xaf1*
Ccl2	*Cdca3*	*F13a1*	*Il10rb*	*Mafb*	*S100a4*	*Zbp1*
Ccl22	*Cdca7*	*Fcer1a*	*Il12a*	*Mcm2*	*S100a6*	*Zfhx3*
Ccl24	*Cdca8*	*Fcrls*	*Il12b*	*Mcm4*	*Sall1*	*Zfp691*
Ccl3	*Cdk1*	*Fen1*	*Il13ra1*	*Mcm5*	*Samhd1*	
Ccl4	*Ceacam1*	*Fgr*	*Il15*	*Mcm6*	*Scd2*	
Ccl5	*Ceacam3*	*Flt3*	*Il17ra*	*Mef2c*	*Sdc1*	
Ccl6	*Cebpa*	*Fos*	*Il18*	*Mki67*	*Sell*	
Ccl7	*Cebpd*	*Fosb*	*Il18bp*	*Mndal*	*Selplg*	
Ccl8	*Cenpa*	*G2e3*	*Il1a*	*Mrc1*	*Siglecf*	
Ccl9	*Cenpe*	*Gas2l3*	*Il1b*	*Ms4a1*	*Siglech*	
Ccnb2	*Cenpf*	*Gas6*	*Il1rn*	*Msh2*	*Slamf7*	
Ccne2	*Chaf1b*	*Gbp2*	*Il2*	*Nampt*	*Slbp*	
Ccr1	*Ckap2*	*Gbp5*	*Il2rb*	*Nasp*	*Slc2a5*	
Ccr2	*Ckap2l*	*Gda*	*Il2rg*	*Ncam1*	*Slc39a1*	
Ccr3	*Ckap5*	*Gins2*	*Il3ra*	*Ncapd2*	*Smc4*	
Ccr7	*Cks1b*	*Gmnn*	*Il4i1*	*Ncoa3*	*Socs1*	
Ccrl2	*Cks2*	*Gpnmb*	*Il5ra*	*Ncr1*	*Socs2*	
Cd14	*Clspn*	*Gpr141*	*Il6*	*Ndc80*	*Socs3*	
Cd163	*Crybb1*	*Gpr34*	*Il6ra*	*Nek2*	*Sparc*	
Cd180	*Csf1*	*Gtse1*	*Irf7*	*Nfia*	*Spp1*	
Cd19	*Cst3*	*H1f0*	*Irf8*	*Nfkbia*	*Tacc3*	
Cd2	*Cst7*	*H1fx*	*Itga2*	*Notch1*	*Tagln2*	
Cd200	*Cstb*	*H2-Aa*	*Itga2b*	*Npc2*	*Tgfbi*	
Cd200r4	*Ctcf*	*H2-Ab1*	*Itga4*	*Nuf2*	*Tgfbr1*	
Cd209a	*Cx3cr1*	*H2-D1*	*Itgam*	*Nusap1*	*Tgm2*	
Cd24a	*Cxcl10*	*H2-DMa*	*Itgax*	*P2ry12*	*Thy1*	

Supplementary Table 4. Specifications, catalog numbers and dilutions of reagents used for immunohistochemistry and flow cytometry.

Reagent	Manufacturer	Cat. number	Clone	Fluorophore	Application	Dilution	Lot number
LiveDead Fixable Violet Dead Cell Stain	ThermoFisher	L34955	-	-	FC	1:1000	1910200
Fixable Viability Dye eF506	eBioscience	65-0866	-	-	FC	1:1000	2198947
Stain Buffer	BD Pharmingen	554656	-	-	FC	-	9329560
Foxp3 Transcription Factor Staining Buffer	eBioscience	00-5523-00	-	-	FC	-	2171417
anti-mouse CD16/CD32 Fc Block	BD Pharmingen	553142	-	-	FC	1:250	8130843
anti-CD45 mAb	BD Pharmingen	561868	30-F11	PE-Cy7	FC	1:800	8205729
anti-CD11b mAb	BD Pharmingen	557960	M1/70	Alexa Fluor 700	FC	1:800	7180930
anti-CD11b mAb	BD Pharmingen	553310	M1/70	FITC	FACS	1:800	8295813
anti-Ly6C mAb	BD Pharmingen	560525	AL-21	PerCP-Cy5.5	FC	1:100	9325105
anti-CD49d mAb	BioLegend	103605	R1-2	FITC	FC	1:400	B239209
anti-PD-L1 mAb	ThermoFisher	63-5982-82	MIH5	SuperBright600	FC	1:100	E113345
anti-Tmem119 mAb	Abcam	ab210405	106-6	unconjugated (rabbit)	FC	1:400	GR3208844-1
anti-rabbit Alexa Fluor 488 pAb	Abcam	ab150077	-	Alexa Fluor 488	FC	1:1000	GR3203087-1
anti-Gal-3 mAb	BioLegend	125408	M3/38	Alexa Fluor 647	FC, IF	1:200 FC<br>1:100 IF	B255908
anti-TMEM119 pAb	Synaptic Systems	400002	-	-	IF	1:500	
anti-rabbit Alexa Fluor 488 pAb	Invitrogen	A21206	-	Alexa Fluor 488	IF	1:1000	1874771

Supplementary References

1. Murphy, K., Janeway, Charles Alderson, J., Travers, P. & Walport, M. J. S. *Janeway's Immunobiology*. (New York : Garland Science, 2012).

2. Sousa, C., Biber, K. & Michelucci, A. Cellular and molecular characterization of microglia: A unique immune cell population. *Front. Immunol.* **8**, (2017).

3. Bennett, M. L. *et al.* New tools for studying microglia in the mouse and human CNS. *Proc. Natl. Acad. Sci. U. S. A.* **113**, E1738–E1746 (2016).

4. Masuda, T. *et al.* Spatial and temporal heterogeneity of mouse and human microglia at single-cell resolution. *Nature* **566**, 388–392 (2019).

5. Matcovitch-Natan, O. *et al.* Microglia development follows a stepwise program to regulate brain homeostasis. *Science* **353**, (2016).

6. Keren-Shaul, H. *et al.* A Unique Microglia Type Associated with Restricting Development of Alzheimer's Disease. *Cell* **169**, 1276-1290.e17 (2017).

7. Bowman, R. L. *et al.* Macrophage Ontogeny Underlies Differences in Tumor-Specific Education in Brain Malignancies. *Cell Rep.* **17**, 2445–2459 (2016).

8. Venteicher, A. S. *et al.* Decoupling genetics, lineages, and microenvironment in IDH-mutant gliomas by single-cell RNA-seq. *Science* **355**, (2017).

9. Müller, S. *et al.* Single-cell profiling of human gliomas reveals macrophage ontogeny as a basis for regional differences in macrophage activation in the tumor microenvironment. *Genome Biol.* **18,** (2017)

10. Walentynowicz, K. A. *et al.* In search for reliable markers of glioma-induced polarization of microglia. *Front. Immunol.* **9**, 1–13 (2018).

11. Haage, V. *et al.* Comprehensive gene expression meta-analysis identifies signature genes that distinguish microglia from peripheral monocytes/macrophages in health and glioma. *Acta Neuropathol. Commun.* **7**, (2019).

12. Jordão, M. J. C. *et al.* Neuroimmunology: Single-cell profiling identifies myeloid cell subsets with distinct fates during neuroinflammation. *Science* **363**, (2019).

13. Goldmann, T. *et al.* Origin, fate and dynamics of macrophages at central nervous system interfaces. *Nat. Immunol.* **17**, 797-805 (2016).

14. Ziegler-Heitbrock, L. *et al.* Nomenclature of monocytes and dendritic cells in blood. *Blood* **116** (2010).

15. Thomas, G. D. *et al.* Human Blood Monocyte Subsets: A New Gating Strategy Defned Using Cell Surface Markers Identifed by Mass Cytometry. *Arterioscler. Thromb. Vasc. Biol.* **37**, 1548–1558 (2017).

16. Fleming, T. J., Fleming, M. L. & Malek, T. R. Selective expression of Ly-6G on myeloid lineage cells in mouse bone marrow. RB6-8C5 mAb to granulocyte-differentiation antigen (Gr-1) detects members of the Ly-6 family. *J. Immunol.* **151**, 2399–408 (1993).

17. Musumeci, A., Lutz, K., Winheim, E. & Krug, A. B. What makes a PDC: Recent advances in understanding plasmacytoid DC development and heterogeneity. *Front. Immunol.* **10** (2019).

18. Crinier, A. *et al.* High-Dimensional Single-Cell Analysis Identifies Organ-Specific Signatures and Conserved NK Cell Subsets in Humans and Mice. *Immunity* **49**, 971-986.e5 (2018).
