# Supplementary material for: Single-cell RNA sequencing reveals functional heterogeneity of glioma-associated brain macrophages
Source: Nat Commun. 2021 Feb 19;12:1151. doi: 10.1038/s41467-021-21407-w (PMC7895824; doi:10.1038/s41467-021-21407-w)
Supplement: Supplementary file 2 — Description of Additional Supplementary Files [file 41467_2021_21407_MOESM2_ESM.pdf]

Description of Additional Supplementary Files

Supplementary Data 1: List of the up to top 30 differentially expressed genes per cluster based on log2 fold change. The lower number of genes means that less than 30 genes were called differentially expressed. Each sheet corresponds to the individual condition as demonstrated in Figure 1b.
