# Supplementary material for: Single-cell RNA sequencing reveals functional heterogeneity of glioma-associated brain macrophages
Source: Nat Commun. 2021 Feb 19;12:1151. doi: 10.1038/s41467-021-21407-w (PMC7895824; doi:10.1038/s41467-021-21407-w)
Supplement: Supplementary file 4 — Reporting Summary [file 41467_2021_21407_MOESM4_ESM.pdf]

nature research

Corresponding author(s): Prof. Bozena Kaminska, Dr Jakub Mieczkowski

Last updated by author(s): 2020/11/03

Reporting Summary

Nature Research wishes to improve the reproducibility of the work that we publish. This form provides structure for consistency and transparency in reporting. For further information on Nature Research policies, see our Editorial Policies and the Editorial Policy Checklist.

Statistics

For all statistical analyses, confirm that the following items are present in the figure legend, table legend, main text, or Methods section.

n/a	Confirmed	
☐	☒	The exact sample size (*n*) for each experimental group/condition, given as a discrete number and unit of measurement
☐	☒	A statement on whether measurements were taken from distinct samples or whether the same sample was measured repeatedly
☐	☒	The statistical test(s) used AND whether they are one- or two-sided<br>*Only common tests should be described solely by name; describe more complex techniques in the Methods section.*
☐	☒	A description of all covariates tested
☐	☒	A description of any assumptions or corrections, such as tests of normality and adjustment for multiple comparisons
☐	☒	A full description of the statistical parameters including central tendency (e.g. means) or other basic estimates (e.g. regression coefficient) AND variation (e.g. standard deviation) or associated estimates of uncertainty (e.g. confidence intervals)
☐	☒	For null hypothesis testing, the test statistic (e.g. *F*, *t*, *r*) with confidence intervals, effect sizes, degrees of freedom and *P* value noted<br>*Give P values as exact values whenever suitable.*
☒	☐	For Bayesian analysis, information on the choice of priors and Markov chain Monte Carlo settings
☐	☒	For hierarchical and complex designs, identification of the appropriate level for tests and full reporting of outcomes
☒	☐	Estimates of effect sizes (e.g. Cohen's *d*, Pearson's *r*), indicating how they were calculated

Our web collection on statistics for biologists contains articles on many of the points above.

Software and code

Policy information about availability of computer code

Data collection	Raw sequencing data (BCL files) were demultiplexed and converted to fastq files using the CellRanger v3.0.1 (10x Genomics) and bcl2fastq v2.20.0.422 (Illumina)
Data analysis	Cell Ranger v3.0.1 count was used to align the demultiplexed reads to the reference genome (mm10), perform filtering, barcode counting, UMI (unique molecule identifier) counting and estimate the number of cells. Further data analysis was performed in R using Seurat v3 package. The source code of the performed analyses and figures described in this study can be found at: github.com/jakubmie/2019_OchockaSegit. Additionally, clusterProfiler v3.12.0 was used for Gene Ontology analysis and scSVA v0.2 (docker image version) was used to create FLE plots.<br>Flow Cytometry data were analyzed with FlowJo software (v. 10.5.3, FlowJo LLC, BD).

For manuscripts utilizing custom algorithms or software that are central to the research but not yet described in published literature, software must be made available to editors and reviewers. We strongly encourage code deposition in a community repository (e.g. GitHub). See the Nature Research guidelines for submitting code & software for further information.

Data

Policy information about availability of data

All manuscripts must include a data availability statement. This statement should provide the following information, where applicable:

- Accession codes, unique identifiers, or web links for publicly available datasets
- A list of figures that have associated raw data
- A description of any restrictions on data availability

scRNA-seq data from performed experiments (BAM files with sequencing output and processed gene expression matrix for each condition) can be downloaded from

the NIH GEO database [GSE136001].
Human glioma scRNA-seq data were downloaded from Tirosh et al. (2016) [GSE70630] and Venteicher et al. (2017) [GSE89567].
For TCGA data analysis the normalized expression values for low- and high-grade gliomas were downloaded from The Cancer Genome Atlas (TCGA) website (RNASeqV2 set available on 07/05/19).

Field-specific reporting

Please select the one below that is the best fit for your research. If you are not sure, read the appropriate sections before making your selection.

- [x] Life sciences
- [ ] Behavioural & social sciences
- [ ] Ecological, evolutionary & environmental sciences

For a reference copy of the document with all sections, see nature.com/documents/nr-reporting-summary-flat.pdf

Life sciences study design

All studies must disclose on these points even when the disclosure is negative.

Sample size	We did not apply statistical tests for sample size calculation. Instead, we decided to use sample sizes similar to those generally employed in the field:<br>Lantz et al. (2020) Nat Comm, 3 pooled animals per sample, 3 samples, 3 conditions, 7 674 cells;<br>Jordao et al. (2019) Science, 54 samples per 12 conditions, 3 461 cells;<br>Masuda et al. (2019) Nature, 3-4 animals per sample group, 28 conditions, 2 966 cells;<br>Sousa et al. (2018) EMBO reports, 2 samples per 2 conditions, 1 247 cells<br><br>We performed scRNAseq using 4 animals per condition (2 samples, pool of 2 animals each) and obtained approximately 5 000 cells per sample, yielding a total of 40 401 cells.
Data exclusions	We did not exclude any samples from scRNA-seq and flow cytometric analyses.
Replication	For scRNA-seq we pooled CD11b+ cells from 2 animals per a replicate to reduce inter animal variability and two replicates obtained this way show good similarity for main subpopulations (Supplementary Figure 2). We performed additional scRNA-seq on naïve (n=2) and sham operated (n=2) animals, and we observed high consistency between replicates.<br>Additionally, we provided the analysis of scRNA-seq data from human gliomas that replicated our finding on sex dependent difference in MHCII expression despite a low number of samples (Figure 5g).<br>For GL261 and primary microglia co-culture experiment (2 biological and 2 technical replicates) all replication attempts were succesfull.
Randomization	For scRNA-seq we designed our experiment to minimize batch effect. All 12 animals were randomly assigned to experimental conditions, and then to pooled samples. Cell sorting was performed in two rounds, in 2 consecutive days. In each round we had 4 samples, 1 from each group: female control, female, tumor, male control and male tumor. Single cell encapsulation and reverse transcription PCR was performed directly after sorting and libraries were prepared after day 2, for all eight samples together.
Blinding	Not relevant. Blinding is used to prevent bias resulted from non-random recruitment or allocation. In our case we used mice from the same source and randomly assigned them to experimental condition.

Reporting for specific materials, systems and methods

We require information from authors about some types of materials, experimental systems and methods used in many studies. Here, indicate whether each material, system or method listed is relevant to your study. If you are not sure if a list item applies to your research, read the appropriate section before selecting a response.

Materials & experimental systems

n/a	Involved in the study	
☐	☒	Antibodies
☐	☒	Eukaryotic cell lines
☒	☐	Palaeontology and archaeology
☐	☒	Animals and other organisms
☒	☐	Human research participants
☒	☐	Clinical data
☒	☐	Dual use research of concern

Methods

n/a	Involved in the study	
☒	☐	ChIP-seq
☐	☒	Flow cytometry
☒	☐	MRI-based neuroimaging

Antibodies

Antibodies used	anti-mouse CD16/CD32 FcBlock BD Pharmingen cat. 553142, (lot: 8130843)<br>anti-CD45 BD Pharmigen cat. 561868, clone 30-F11, PE-Cy7 (lot: 8205729)<br>anti-CD11b BD Pharmigen cat. 557960, clone M1/70, AF700 (lot: 7180930)

anti-CD11b BD Pharmigen cat. 553310, clone M1/70, FITC (lot: 8295813)
anti-Ly6C BD Pharmigen cat. 560525, clone AL-21, PerCP-Cy5.5 (lot: 9325105)
anti-CD49d BioLegend cat. 103605, clone R1-2, FITC (lot: B239209)
anti-PD-L1 ThermoFisher cat. 63-5982-82, clone MIH5, SuperBright600 (lot: E113345)
anti-Tmem119 Abcam cat. ab210405, clone 106-6, unconjugated (rabbit) (lot: GR3208844-1) - used for FC
anti-rabbit pAb Abcam cat. ab150077, AF488 (lot: GR3203087-1) - used for FC
anti-Gal-3 BioLegend cat. 125408, clone M3/38, AF647 (lot: B255908)
anti-Tmem119 SynaptcSystems cat 400002, unconjugated - used for IF
anti-rabbit pAb Invitrogen cat. A21206, AF488 (lot: 1874771) - used for IF

Validation

Flow cytometry antibodies conjugated with fluorochromes utilized in this study are commonly used and have been validated in previous studies/by manufacturers, especially by staining cells serving as positive/negative biological controls for specific receptors. Unconjugated antibody against Tmem119 (Abcam cat. ab210405) has been initially validated by group that has developed it, by use of knock-out animals (Bennet et al. 2016). Each primary antibody was titrated prior to use to establish an optimal concentration for a specific staining.

Eukaryotic cell lines

Policy information about cell lines

Cell line source(s)

GL261 was obtained from Prof. Helmut Kettenmann (MDC, Berlin, Germany) and originally purchased from The Jackson Laboratory. GL261 tdT+ luc+ - was developed from GL261

Authentication

We did not perform cell line authentication.

Mycoplasma contamination

The cell lines tested negative for mycoplasma contamination prior to cryopreservation.

Commonly misidentified lines
(See ICLAC register)

None of the used cell liness are placed in the ICLAC register.

Animals and other organisms

Policy information about studies involving animals; ARRIVE guidelines recommended for reporting animal research

Laboratory animals

C57BL6 mice, 10 weeks old, male and female, purchased from the Medical University of Bialystok, Poland.

Wild animals

The study did not involve wild animals

Field-collected samples

The study did not involve samples collected in the field.

Ethics oversight

All experimental procedures on animals were approved by the First Local Ethics Committee for Animal Experimentation in Warsaw (approval no 563/2018 and 764/2018).

Note that full information on the approval of the study protocol must also be provided in the manuscript.

Flow Cytometry

Plots

Confirm that:

- [x] The axis labels state the marker and fluorochrome used (e.g. CD4-FITC).
- [x] The axis scales are clearly visible. Include numbers along axes only for bottom left plot of group (a 'group' is an analysis of identical markers).
- [x] All plots are contour plots with outliers or pseudocolor plots.
- [x] A numerical value for number of cells or percentage (with statistics) is provided.

Methodology

Sample preparation

The tumor-bearing hemispheres and corresponding hemispheres from naïve animals were dissociated enzymatically to obtain a single-cell suspension with a Neural Tissue Dissociation Kit with papain (Miltenyi Biotec) or 0.5 mg/mL DNase I (DN25, Sigma-Aldrich) and gentleMACS Octo Dissociator (Miltenyi Biotec). Myelin was removed by centrifugation on 22% Percoll gradient. Next, cells were quantified using an EVE™ Automatic Cell Counter (NanoEnTek Inc., USA), and split for CD11b + FACS and cytometric analysis.

Instrument

For FACS, samples were sorted using BD FACSAria II. For phenotypic cytometry analysis, samples were acquired using a BD LSR Fortessa Analyzer.

Software

Data were analyzed with FlowJo software (v. 10.5.3, FlowJo LLC, BD).

Cell population abundance	For FACS, 200 000 cells were sorted with over 96% purity as assessed by post-sort analysis. For phenotypic cytometry analysis, on average around 35 000 viable CD11b+ events were acquired for further analysis.
Gating strategy	Singlets were analyzed for the uptake of either Fixable Viability Dye or LiveDead Violet dye to exclude events corresponding to dead or apoptotic cells. For the gating of Tmem119+ and Gal-3+ events, gates were set on CD11b+ events. For CD49d+ and PD-L1+ events, gates were set on CD11b+ events. For Ly6C vs CD49d and Ly6C vs PD-L1 analysis, gates were set on CD11b +CD45hi events. Gates were set based on backgating strategy or FMO controls.

☒ Tick this box to confirm that a figure exemplifying the gating strategy is provided in the Supplementary Information.
